# Psychomotor and non-motor correlates of cognition in spinocerebellar ataxias Types 1, 2, 3, and 6

**DOI:** 10.1093/braincomms/fcaf425

**Published:** 2025-10-28

**Authors:** Louisa P Selvadurai, Sheryl Gullia, James Morgan, Sarah Wallis, Kishore R Kumar, David J Szmulewicz, Ian H Harding

**Affiliations:** Department of Neuroscience, School of Translational Medicine, Monash University, Melbourne, Victoria 3004, Australia; School of Psychological Sciences, Monash University, Clayton, Victoria 3800, Australia; School of Psychological Sciences, Monash University, Clayton, Victoria 3800, Australia; Department of Neuroscience, School of Translational Medicine, Monash University, Melbourne, Victoria 3004, Australia; Department of Neuroscience, School of Translational Medicine, Monash University, Melbourne, Victoria 3004, Australia; School of Psychological Sciences, Monash University, Clayton, Victoria 3800, Australia; Molecular Medicine Laboratory and Department of Neurology, Concord Repatriation General Hospital, Concord Clinical School, University of Sydney, Concord, NSW 2139, Australia; Genomics and Inherited Disease Program, Garvan Institute of Medical Research, Darlinghurst, NSW 2010, Australia; School of Clinical Medicine, UNSW Medicine & Health, University of New South Wales, Kensington 2033, NSW, Australia; Balance Disorders & Ataxia Service, Royal Victorian Eye and Ear Hospital, East Melbourne, Victoria 3002, Australia; NeuroMovement Laboratory, Bionics Institute, Fitzroy, Victoria 3065, Australia; Department of Neuroscience, School of Translational Medicine, Monash University, Melbourne, Victoria 3004, Australia; QIMR Berghofer Medical Research Institute, Herston, Queensland 4006, Australia

**Keywords:** spinocerebellar ataxia, cerebellar cognitive affective syndrome, cognition, cognitive assessment, ataxia

## Abstract

There is growing evidence of cognitive deficits in spinocerebellar ataxias, with the Cerebellar Cognitive Affective Syndrome Scale (CCAS-S) an increasingly common measure of this dysfunction. There remain ongoing questions as to how Cerebellar Cognitive Affective Syndrome Scale performance relates to day-to-day cognitive function, non-motor and motor features of spinocerebellar ataxias and demographic factors. Via an online study, we evaluated Cerebellar Cognitive Affective Syndrome Scale performance amongst individuals with spinocerebellar ataxia Type 1 (n = 14), Type 2 (n = 16), Type 3 (n = 18), and Type 6 (n = 26) relative to demographically-matched control groups. Furthermore, amongst individuals with spinocerebellar ataxia, we examined associations between performance and (i) age and education, (ii) ataxia motor severity, (iii) psychomotor function measured by computerized finger tapping and reaction time tasks and (iv) self-rated cognition, depression, emotional regulation, psychosocial function and fatigue. Cerebellar Cognitive Affective Syndrome Scale performance was significantly reduced in spinocerebellar ataxia Types 2, 3, and 6 compared to controls, although substantial inter-individual variability in performance was observed in the spinocerebellar ataxia cohort (43.2%/24.3%/21.6%/10.8% met criteria for Definite, Probable, Possible, and No CCAS). Performance in individuals with spinocerebellar ataxias correlated significantly with self-reported ataxia motor severity, fine motor speed, psychomotor trial-by-trial variability, and one of two measures of day-to-day cognitive function. Significant correlations were not observed against age, education, age at disease onset, disease duration, psychomotor reaction time, depression, emotional regulation, psychosocial function, or fatigue. We present evidence that motor function and psychomotor variability are more important correlates of inter-individual variability in cognitive performance amongst people with spinocerebellar ataxia Types 1, 2, 3 and 6, compared to demographic factors, fatigue, or emotional function. Importantly, formalized cognitive testing using the Cerebellar Cognitive Affective Syndrome Scale correlates with self-reported cognitive functioning. This study highlights cognitive dysfunction as a functionally impactful feature of certain spinocerebellar ataxias, and motivates further investigation into the disease- and individual-specific profiles of cognitive impairment in this population.

## Teaser

Selvadurai *et al*. report that individuals with spinocerebellar ataxias (SCAs) 2, 3, and 6 have reduced average cognitive screening performance relative to controls. They also demonstrate that cognitive performance in SCAs is associated with self-reported day-to-day cognition, as well as self-reported motor function and psychomotor variability.

## Graphical Abstract

**Graphical Abstract:**
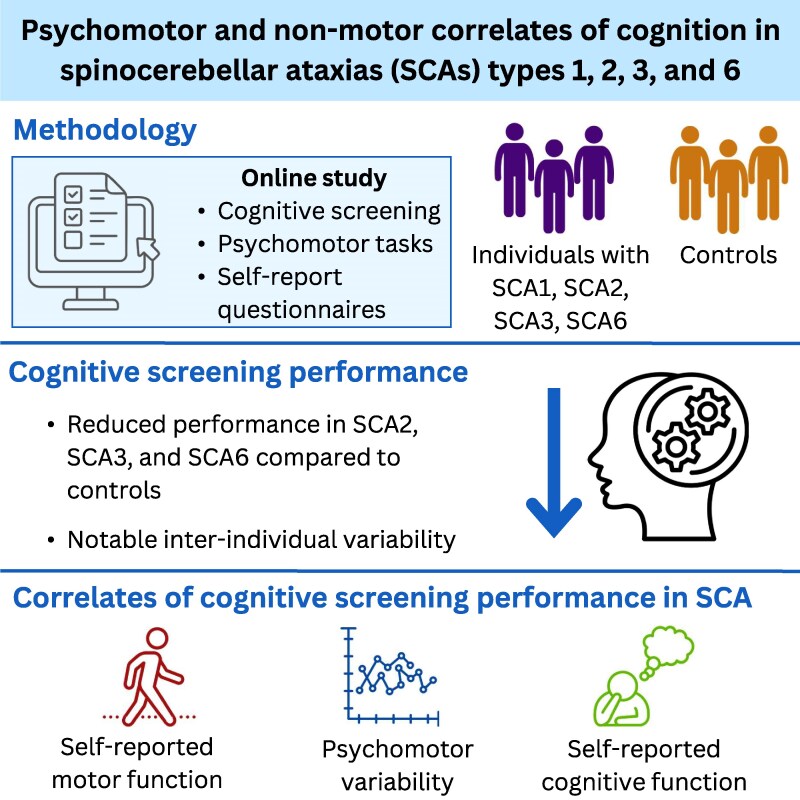

## Introduction

Spinocerebellar ataxias (SCAs) are dominantly-inherited, progressive, and functionally disabling neurodegenerative disorders. The most common SCA types include SCA1, SCA2, SCA3, and SCA6, caused by CAG tandem repeats in the *ATXN1*, *ATXN2*, *ATXN3*, and *CACNA1A* genes, respectively.^1^ SCAs involve damage to the cerebellum and cerebellar pathways.^1^ These structures have a regulatory role in motor activity,^2^ and their disruption leads to ataxia (movement incoordination) in SCA, a characteristic feature amongst other symptoms which implicate various functions and body systems (e.g. sleep disorder, vestibular dysfunction, parkinsonism, tremor, and fatigue)^3-6^. Furthermore, the cerebellum and its pathways have a role in the regulation of cognition and emotion; the disruption of these functions is referred to as the Cerebellar Cognitive Affective Syndrome (CCAS), whereby damage to the posterior cerebellum produces difficulties in executive, visuospatial, linguistic, and affective function.^7^ In line with this, reduced cognitive performance is observed in SCAs, in at least some individuals.^8^ Cognitive deficits must be identified and managed in individuals with SCA to maximize quality of life in the context of the progressive and debilitating motor symptoms.

The Cerebellar Cognitive Affective Syndrome Scale (CCAS-S) was introduced in 2018^9^ as a screening instrument for CCAS amongst individuals with a known cerebellar disorder. The CCAS-S contains 10 domains evaluating performance in different cognitive functions. Application of the CCAS-S to various mixed and single-diagnosis SCA cohorts has revealed reduced average performance relative to controls.^10-16^ The CCAS-S is therefore a useful cognitive screening tool in these conditions.

Nevertheless, not all individuals with SCA demonstrate cognitive difficulties on the CCAS-S. With respect to demographic factors potentially underlying these inter-individual differences, educational attainment has been identified as a consistent correlate of CCAS-S performance, while age is less strongly and less consistently related.^10-12,14^ Furthermore CAG triplet repeat length, age at ataxia onset, and disease duration show limited correlations with CCAS-S outcomes.^10-12,16^ Further investigation is needed to understand demographic, environmental, and disease history factors underlying inter-individual differences in CCAS-S performance.

Research also supports a relationship between cognitive performance and other aspects of SCA disease expression. This includes correlations between poorer CCAS-S performance and worse performance on motor tasks such as the Scale for the Assessment and Rating of Ataxia (SARA), the 9-Hole Peg Test, and timed walking tests.^10-12,16^ However, it is not well understood whether CCAS-S performance relates to a more diverse outcomes, including day-to-day cognitive function. This exposes a crucial deficit in our understanding of the scale’s ecological validity and the real-world functional relevance of cognitive symptoms.

There is a need to better understand CCAS-S performance as it relates to other outcome measures. The relative rarity of SCAs raises challenges for obtaining adequate study cohorts via centre-based studies. Decentralized approaches, including internet-based remote assessments, can offer benefits in this context. In remote study designs, participants can interact with researchers via teleconferencing, and/or complete online assessments independently. This eliminates the time, effort, cost, and carer burden of travelling to a research centre,^17^ particularly for individuals with conditions such as SCA which are associated with impaired mobility and fatigue. Remote assessments can also improve access to more diverse cohorts^18^ and can allow for more frequent data collection than is possible within in-person designs. On the other hand, this approach also has limitations. This includes less control over the testing environment including performance differences in hardware and web-browsers used by participants.^17,19^ Furthermore, there is greater burden on participants to navigate the study tasks,^18^ and the potential for non-valid completion of unsupervised assessments. Other challenges include potential bias towards individuals with greater digital proficiency and access,^20^ and the inability to conduct certain assessments such as physical examinations. Nevertheless, the benefits and reduced participant burden associated with remote, online study designs indicate that they hold promise for progressing research in SCAs.

In this study, we investigated CCAS-S performance amongst individuals with SCA 1, 2, 3, and 6, and demographically-matched control cohorts using an internet-based remote assessment approach. We aimed to first assess CCAS-S performance in SCAs relative to controls and evaluate its relationship to demographic factors and ataxia severity, as a replication of previous work. We then evaluated associations between CCAS-S performance, and both performance level and variability on objective psychomotor tasks. Finally, we examined associations between CCAS-S performance and self-reported measures of non-motor function including cognition, psychosocial function, and fatigue.

## Materials and methods

### Participants

This study forms part of the SCA-Remote study undertaken at Monash University, Australia. Approval was obtained from the Human Research Ethics Committee (HREC) of Monash University (Ethics approval number 26568). The procedures used in this study adhere to the tenets of the Declaration of Helsinki. Written informed consent was obtained from all individual participants included in the study.

Adults (aged ≥ 18) with symptomatic SCA1 (*n* = 14), SCA2 (*n* = 16), SCA3 (*n* = 18), and SCA6 (*n* = 26), and adult control participants without SCA (*n* = 57), were recruited via internal participant databases, patient advocacy organizations, social media, and word-of-mouth. Participants who completed an online expression of interest were invited to participate if they met eligibility criteria.

Inclusion criteria included fluent English speaking and reading, and access to a computer with microphone and internet access, but was not limited by geographic location. Participants with SCA had a molecular diagnosis of SCA. Exclusion criteria for individuals with SCA included (i) diagnosis of an additional neurological condition (including an acquired brain injury) and (ii) diagnosis of a psychiatric condition (other than clinical depression and/or general anxiety). Individuals with SCA who reported a depression or anxiety diagnosis were retained because elevated depression and anxiety symptoms are observed as part of the symptom profile.^21^ Exclusion criteria for Control participants included diagnosis of any neurological or psychiatric condition (including a diagnosis of depression and/or anxiety).

A demographically-matched control group was objectively selected from the full control cohort for each SCA type based on age, sex, and years of education, using the MatchIt package in R with 1:1 nearest neighbour matching on propensity score (see Supplementary Table 1 for match statistics). Demographic and clinical characteristics of the matching groups are presented in Table 1. Data include in the analyses were collected between May 2021 and August 2023. Participants were located in the following countries: Australia, Brazil, Canada, Finland, Germany, India, Isle of Man, South Africa, UK, and USA.

**Table 1:** Demographic and clinical information for SCA and matched control groups

	SCA1	CON1	SCA2	CON2	SCA3	CON3	SCA6	CON6
N	14	14	16	16	18	18	26	26
**Demographic data**								
Age (years)	45.14 ± 11.63	47.86 ± 15.93^a^	55.12 ± 12.63	55.12 ± 13.99^a^	46.5 ± 11.8	47.28 ± 12.05^a^	62.12 ± 11.46	60.73 ± 12.09^a^
Sex	10F; 4M	9F; 5M^a^	9F; 7M	10F; 6M^a^	11F; 7M	10F; 8M^a^	13F; 13M	15F; 11M^a^
Years of education	16.43 ± 2.95	16.14 ± 4.04^a^	17.69 ± 2.75	18.31 ± 2.7^a^	16.94 ± 2.96	17.33 ± 3.38^a^	14.5 ± 4.58	15.27 ± 3.31^a^
**Clinical data**								
Age at disease onset (years)^b^	39.36 ± 10.58	-	39.93 ± 12.58 (*n* = 15)	-	37.22 ± 9.92	-	51.77 ± 12.33	-
Disease duration (years)	5.79 ± 3.26	-	14.47 ± 7.49 (*n* = 15)	-	9.28 ± 4.96	-	10.35 ± 8.4	-
Functional staging of ataxia score (0–6)	2.36 ± 0.74	-	2.5 ± 1.1	-	3.22 ± 1.4	-	2.69 ± 1.09	-
**Met criteria for definite CCAS (3 + failed CCAS-S items)—n (%)**								
Yes	5 (35.7%)	2 (14.3%)	7 (43.8%)	3 (18.8%)	8 (44.4%)	4 (22.2%)	12 (46.2%)	3 (11.5%)
No	9 (64.3%)	12 (85.7%)	9 (56.3%)	13 (81.3%)	10 (55.6%)	14 (77.8%)	14 (53.8%)	23 (88.5%)

CON1 = matched control group for the SCA1 cohort. Values are mean ± standard deviation.

^a^Control group is statistically matched with the relevant SCA group (group comparison *P* > 0.05; see Supplementary Table 1 for statistical data).

^b^Onset of motor symptoms of ataxia.

### Materials

Participants completed the following assessments remotely on a personal computer with researcher guidance via Zoom teleconferencing.

#### Demographic and clinical information

Participants completed an online demographic and clinical information survey. This included self-reported age, sex, and years of education (all education including primary, secondary, and tertiary). Age at ataxia onset was defined as the age at first manifestation of motor symptoms, based on participant self-report per other SCA natural history studies.^12,13^ If an onset age range was reported, the earliest age was used. Ataxia disease duration was calculated as current age minus age at ataxia onset.

Participants with SCA completed self-reported measures of ataxia motor severity, including the Functional Staging for Ataxia, a 7-point scale from the Friedreich Ataxia Rating Scale (FARS),^22^ and the ‘Physical’ and ‘Activities of Daily Living (ADLs)’ subscales of the 70-item Patient-Reported Outcome Measure of Ataxia (PROM-Ataxia).^23^ Higher scores on all scales indicate greater symptom severity.

#### The CCAS-S

Version A of the CCAS-S^9^ was administered by a researcher, with modifications to accommodate the teleconferencing medium (Supplementary Table 2). The CCAS-S contains 10 domains: semantic fluency, phonemic fluency, category switching, digit span forwards, digit span backwards, cube drawing/copying, verbal recall, similarities, go no-go, and affect. Each domain generates a raw score and a pass/fail designation based on a cut-off, producing a total raw score (0–120) and total number of failed domains (fail score; 0–10). Higher raw scores indicate better cognitive performance. Furthermore, the presence of CCAS is categorized based on failed domains: possible (1), probable (2) or definite (3 or more).

#### Psychomotor measures

Participants completed a series of online psychomotor tasks via Inquisit Web (2021).^24^

- *Speeded finger tapping*: Tapping the spacebar as quickly as possible with the nondominant index finger for 10 s, repeated 3 times. The median and median absolute deviation (MAD; variability) of inter-tap intervals were calculated.
- *Paced finger tapping*: Alternately pressing the ‘A’ and ‘L’ keyboard keys with left and right index fingers, respectively, in time with a tone presented every 555 milliseconds. After 10 tones, the paced tone stopped and participants continued tapping at the same time rate for an additional 20 taps. The task was repeated 3 times. The median and MAD of the non-paced inter-tap intervals were calculated.
- *Visual reaction time*: Focussing on a fixation cross and pressing the spacebar as quickly as possible upon presentation of a central red circle on the screen. Stimuli were presented 20 times, at random delay intervals ranging from 2–8 s.
- *Arrow flanker*: Presentation of a horizontal line of 5 arrows each pointing to the left or the right. In ‘congruent’ trials, the arrows all pointed in the same direction; in ‘incongruent’ trials the central arrow pointed in the opposite direction to the other arrows. Participants indicated the central arrow direction using the ‘A’ (left) and ‘L’ (right) keyboard keys. Sixteen congruent and 16 incongruent trials were presented.

Median and MAD of response latencies were calculated for Visual Reaction Time, Arrow Flanker congruent, and Arrow Flanker incongruent. ‘Decision time’ median and MAD were calculated as the difference between Arrow Flanker congruent and Visual Reaction Time median and MAD, respectively. ‘Inhibition time’ median and MAD were calculated as the difference between Arrow Flanker incongruent and Arrow Flanker congruent median and MAD, respectively.

Median and MAD are measures of central tendency and variability that are robust to outliers and non-normal data distributions.

#### Subjective non-motor measures

Participants completed self-report questionnaires from the NIH Neuro-QoL (Quality of Life in Neurological Disorders) battery.^25^ Using the following 8-item surveys, participants rated their subjective function in the past seven days: Cognitive Function; Depression; Emotional and Behavioural Dyscontrol; Fatigue. Higher total raw scores (8–40) indicate better function on the Cognitive Function scale and poorer function on all other scales.

Participants with SCA also completed the Mental 1 (psychosocial), and Mental 2 (cognitive) domains of the PROM-Ataxia; higher scores reflect poorer outcomes.

### Statistical analyses

REDCap electronic data capture tools hosted at Monash University provided survey delivery and data management.^26-29^ Data were exported to RStudio (2024.04.2) for analysis using R software (version 4.4.1). Statistical significance was evaluated at *P* < 0.05.

#### Group comparisons

The proportion of individuals in each group meeting criteria for the categories of No, Possible, Probable, and Definite CCAS was calculated.

CCAS-S total raw scores, fail scores, and individual domain scores were compared between each SCA type and its matched control group using non-parametric Mann-Whitney *U* tests and rank biserial *r* effect sizes, due to the presence of non-normal data distributions.

#### Correlation analyses

Spearman correlations were used to evaluate associations between each of CCAS-S total raw score and total fail score, and (i) demographic variables, (ii) ataxia severity and clinical measures, (iii) psychomotor task median performance and performance variability (median absolute deviation) and (iv) subjective non-motor questionnaire scores. Correlations were conducted across the combined SCA cohort. Demographic correlations were additionally conducted within the combined Control group.

We also conducted a supplementary multiple regression analysis to evaluate the relative contribution of motor function and cognitive function to the verbal fluency components of the CCAS-S (semantic fluency, phonemic fluency, and category switching scores combined).

## Results

### CCAS categorization

Across all SCA types (*n* = 74), 43.2% of individuals met criteria for Definite CCAS. Across all controls (n = 57), 14.0% met criteria for Definite CCAS. CCAS categories within each participant group is presented in Table 1. Supplementary Fig. 1 displays CCAS category membership as proportions within each SCA type, the combined SCA group, and the full Control group.

### Group comparisons

**Figure 1:**
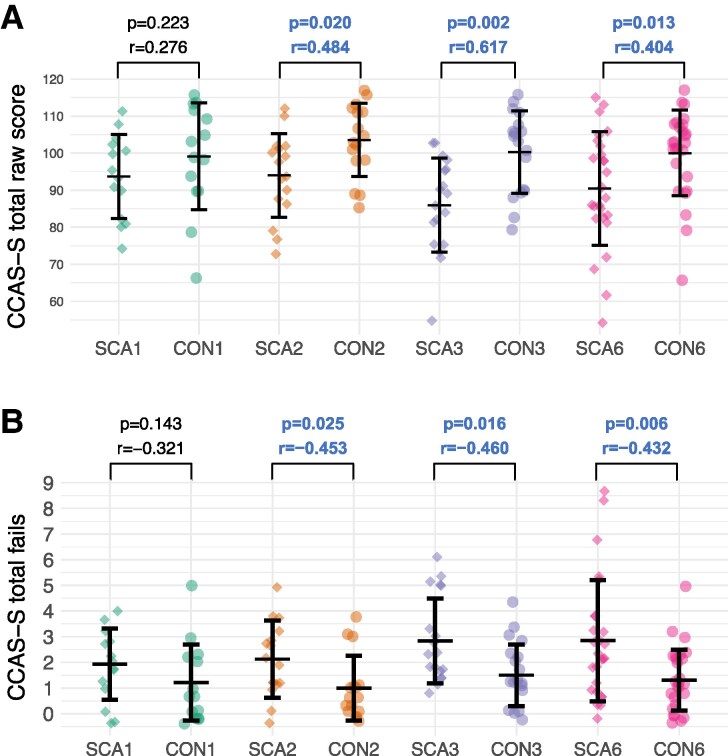
**Differences between spinocerebellar ataxia cohorts and matched control groups on the CCAS-S.** (**A**) CCAS-S total raw score (**B**) CCAS-S total fails. Each datapoint represents a single participant. Sample sizes: SCA1: *n* = 14; CON1: *n* = 14; SCA2: *n* = 16; CON2: n = 16; SCA3: *n* = 18; CON3: n = 18; SCA6: *n* = 26; CON6: *n* = 26. Comparisons are Mann-Whitney *U* tests, with significant results at *P* < 0.05 shown in bold and blue text. Effect size is indicated by point biserial *r*. SCA1 = spinocerebellar ataxia type 1 group. CON1 = control group matched to the SCA1 group.

Non-parametric Mann-Whitney *U* tests demonstrated significantly lower CCAS-S total raw scores (Fig. 1A), and higher CCAS-S total fail scores (Fig. 1B), in the SCA2, SCA3, and SCA6 groups relative to their matched control groups, with moderate to large effects. Group differences between the SCA1 group and its control group were not statistically significant (small to moderate effects).

**Figure 2:**
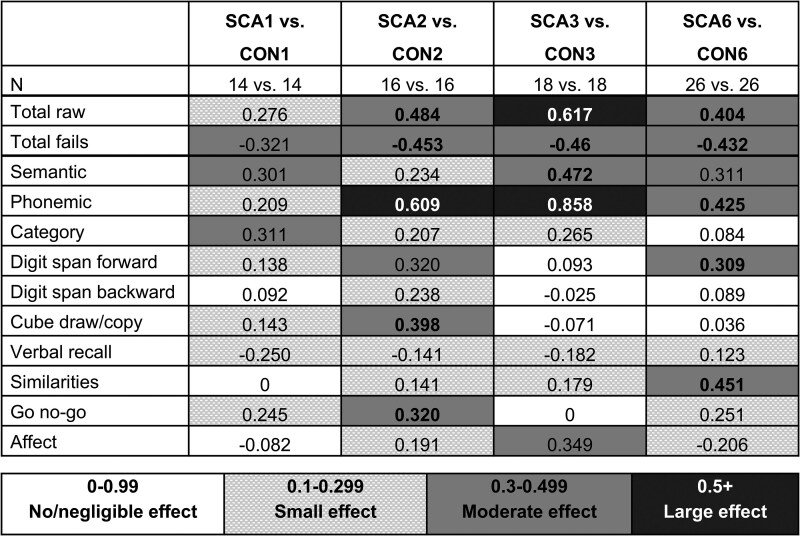
**Effect sizes for SCA-control comparisons across domains on the CCAS-S.** Point biserial *r* effect sizes for CCAS-S total and domain comparisons between the SCA and Control groups. SCA1 = spinocerebellar ataxia type 1 group. CON1 = control group matched to the SCA1 group. Positive effect size value means SCA < CON; negative effect size value means SCA > CON. Bold text indicates that the comparison was statistically significant at *P* < 0.05. Effect size colour guide shown beneath grid. CCAS-S = Cerebellar Cognitive Affective Syndrome Scale.

Supplementary Tables 3–6 provide descriptive statistics for CCAS-S total raw score and fail scores in each SCA type and matched control group, and present statistics for group comparisons within each CCAS-S domain. Group difference effect sizes across domains are calculated only for exploratory presentation of shared and unique trends between SCA types (Fig. 2), as this study is not statistically powered to undertake statistical inference of between-group differences with proper multiple comparison correction. Notably, large group differences were observed for phonemic fluency, in the SCA2 and SCA3 groups, and larger effects were observed for phonemic fluency compared to semantic fluency in SCA2, SCA3, and SCA6. Apart from this observation, there did not appear to be a shared profile of effect sizes across domains between SCA types. For example, there were instances of a moderate group difference effect in one SCA type but negligible or small effects in others: cube draw/copy and go no-go in SCA2, similarities in SCA6, and affect in SCA3. These preliminary patterns should be replicated in larger samples, and should be interpreted with caution here due to non-equivalence of participant characteristics between the SCA genotype cohorts.

### CCAS-S correlates

Scatterplots and statistics for CCAS-S raw score correlations are displayed in the main text, and those for fail scores in the Supplementary Materials.

#### Demographic and clinical variables

**Figure 3:**
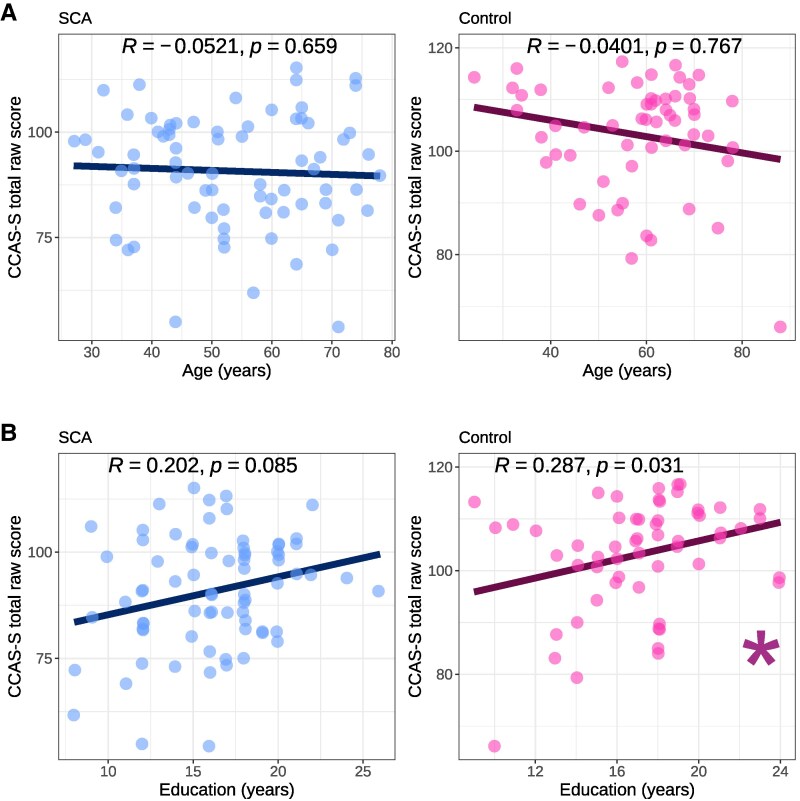
**CCAS-S raw score versus demographic variables.** Scatterplots showing the relationship between CCAS-S total raw score and (**A**) age and (**B**) years of education in the combined SCA (*n* = 74) and Control (*n* = 57) groups. Each datapoint represents a single participant. *R* = Spearman correlation coefficient. Asterisk indicates a significant correlation at *P* < 0.05. CCAS-S = Cerebellar Cognitive Affective Syndrome Scale; SCA = spinocerebellar ataxia.

More years of education was significantly associated with higher CCAS-S total raw score in the Control group, and was trending at *P* < 0.10 in the combined SCA group. Education years were not significantly related to fail score. No significant associations were observed between age and CCAS-S performance in either the SCA or Control groups. See Fig. 3 (CCAS-S raw scores) and Supplementary Fig. 2 (CCAS-S fail scores) for scatterplots and correlation statistics.

**Figure 4:**
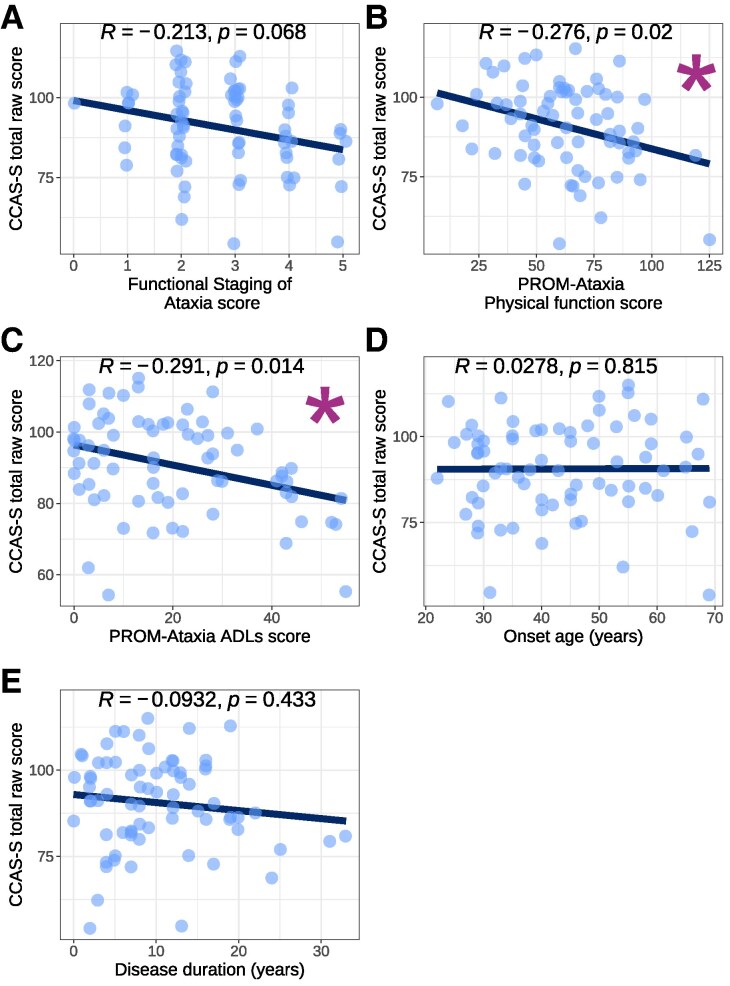
**CCAS-S raw score versus clinical variables.** Scatterplots showing the relationship between CCAS-S total raw score and clinical variables: (**A**) Functional Staging of Ataxia score (*n* = 74); PROM-Ataxia (**B**) Physical function and (**C**) ADLs (*n* = 71), (**D**) onset age (*n* = 73) and (**E**) disease duration (*n* = 73). Each datapoint represents a single participant. *R* = Spearman correlation coefficient. Asterisk indicates a significant correlation at *P* < 0.05. CCAS-S = Cerebellar Cognitive Affective Syndrome Scale; PROM-Ataxia = Patient-Reported Outcome Measure of Ataxia; ADLs = Activities of Daily Living.

Lower CCAS-S raw scores were significantly associated with greater disease severity with small to moderate effects, indexed by the PROM-Ataxia Physical Function and ADL scales (Fig. 4), with a trending association at *P* < 0.10 for Functional Staging of Ataxia. Similarly, small to moderate significant associations were observed between a greater number of failed domains and greater disease severity (Supplementary Fig. 3). Neither onset age nor disease duration correlated with CCAS-S performance (Fig. 4, Supplementary Fig. 3).

Genetic repeat length data (number of CAG repeats on the relevant gene) was only collected for a small subset of participants with SCA (*n* = 28), precluding meaningful correlation analyses within genotypes. However, associations are shown in Supplementary Fig. 4 for completeness. Furthermore, we used CAG repeat length to calculate a ‘genetic burden’ value that quantified repeat length as excess repeat length relative to a non-expanded repeat length, as a proportion of the non-expanded repeat length, per our previous work.^30^ This value can be compared across genotypes. We observed non-significant and small correlations between genetic burden value and CCAS-S performance (see Supplementary Fig. 5).

#### Psychomotor performance

**Figure 5:**
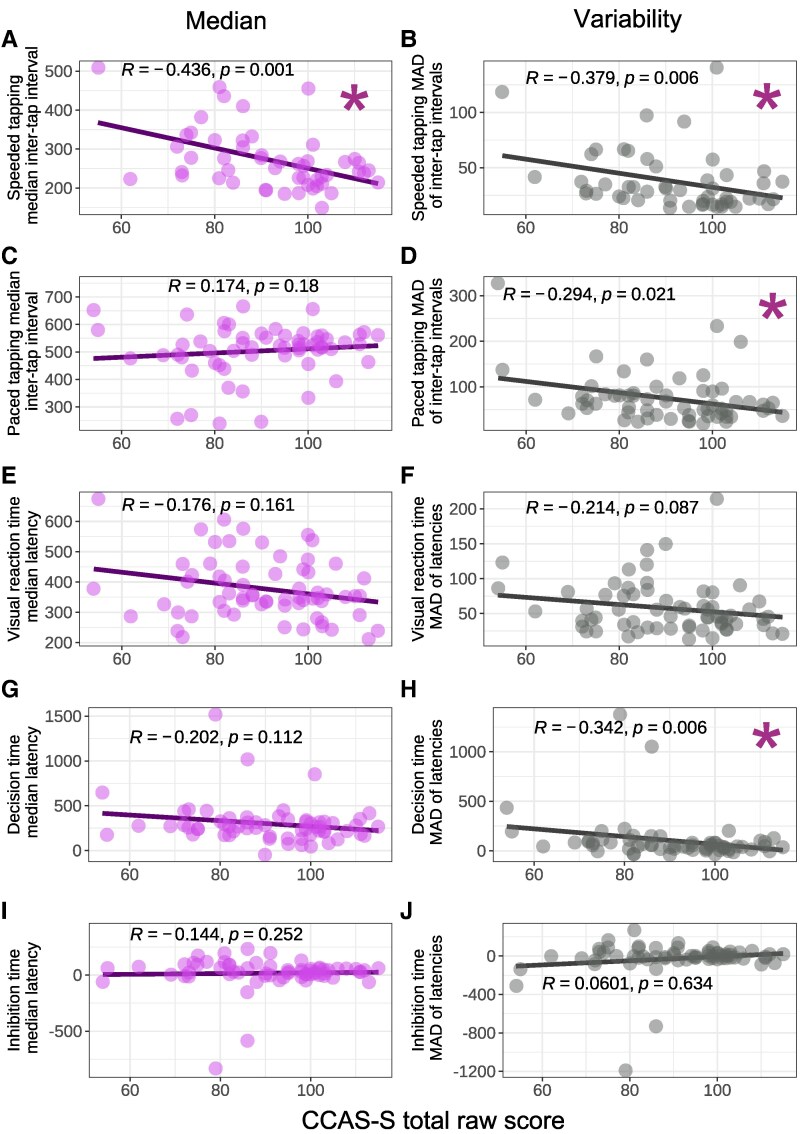
**CCAS-S raw score versus psychomotor variables.** Scatterplots showing the relationship between CCAS-S total raw score and median (left column) and median absolute deviation (MAD; right column) performance on psychomotor tasks: (A-B) speeded tapping (*n* = 51), (C-D) paced tapping (*n* = 61), (E-F) visual reaction time (*n* = 65), (G-H) decision time (*n* = 63) and (I-J) inhibition time (*n* = 65). Each datapoint represents a single participant. *R* = Spearman correlation coefficient. Asterisk indicates a significant correlation at *P* < 0.05. CCAS-S = Cerebellar Cognitive Affective Syndrome Scale; MAD = median absolute deviation; measure of variability.

Correlations were conducted amongst SCA participants with valid data for each psychomotor task. Descriptive statistics for the psychomotor task outcomes are presented in Supplementary Table 7. Missing data is due to task non-completion, for example due to technical difficulties. Figure 5 and Supplementary Fig. 6 display scatterplots and correlation statistics.

Median speed and variability (MAD) on speeded tapping were both significantly correlated with CCAS-S total raw and total fail score, with moderate to large effects. Variability in paced tapping and decision time were significantly associated with total raw and fail scores, with small to moderate effects.

#### Subjective non-motor function

Poorer function on the PROM-Ataxia Mental 2 (cognitive) domain was significantly associated with lower CCAS-S total score and more failed domains, with moderate effects. A trending correlation (*P* < 0.1) was observed between Neuro-QoL depression and total fail score.

**Figure 6:**
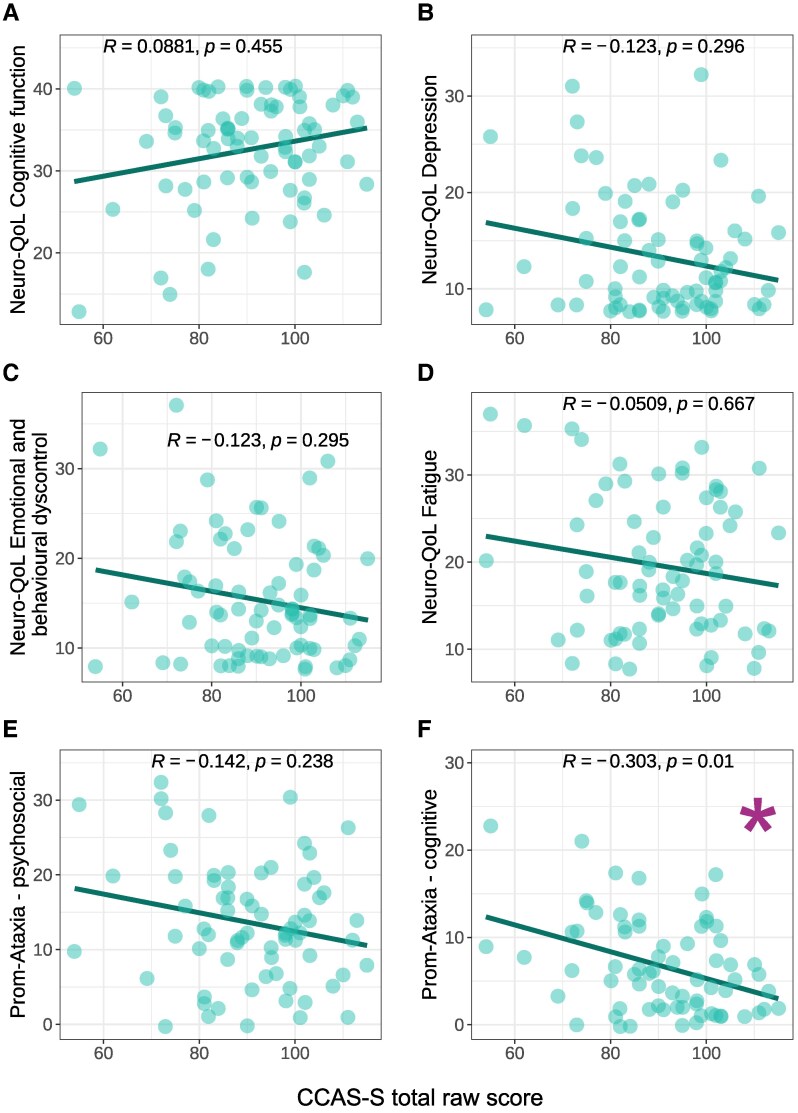
**CCAS-S raw score versus subjective non-motor variables.** Scatterplots showing the relationship between CCAS-S total raw score and subjective non-motor outcomes; Neuro-QoL scales (*n* = 74)—**(A)** Cognitive function, (**B**) Depression, (**C**) Emotional and behavioural dyscontrol, (**D**) Fatigue; PROM-Ataxia (*n* = 71)—**(E)** Mental 1 (psychosocial) and **(F)** Mental 2 (cognitive). Each datapoint represents a single participant. *R* = Spearman correlation coefficient. Asterisk indicates a significant correlation at *P* < 0.05. CCAS-S = Cerebellar Cognitive Affective Syndrome Scale; Neuro-QoL = Quality of Life in Neurological Disorders; PROM-Ataxia = Patient-Reported Outcome Measure of Ataxia.

Scatterplots and correlation statistics are displayed in Fig. 6 and Supplementary Fig. 7. Supplementary Table 8 provides non-motor variable descriptive statistics.

Raw scores from the semantic fluency, phonemic fluency, and category switching domains were summed to produce a CCAS-S ‘verbal fluency’ score. A multiple linear regression was used to evaluate the relative contributions of self-reported Physical function (PROM-Ataxia Physical score) and Cognitive function (PROM-Ataxia Mental 2 score) to the verbal fluency score. Together, Physical and Cognitive function significantly predicted verbal fluency score, *F*(2,68) = 5.19, *P* < 0.008. When considered independently (i.e. controlling for variance associated with the other predictor), Cognitive function was a stronger predictor (β = −0.504, *P* = 0.073) than Physical function (β = −0.064, *P* = 0.330), although neither reached statistical significance alone (see Supplementary Table 9 for further statistics).

Exploratory correlations between CCAS-S performance and all outcome measures, within each SCA genotype, are presented in Supplementary Table 10.

## Discussion

We aimed to investigate the pattern and correlates of cognitive performance in SCA1, 2, 3, and 6, via online administration of the CCAS-S. We report reduced CCAS-S performance in SCA2, SCA3, and SCA6 relative to matched control groups, and associations between CCAS-S performance and both education level and ataxia severity. Furthermore, we present additional CCAS-S correlates including objective psychomotor performance and variability, and self-rated cognitive function.

### CCAS-S performance in SCA cohorts

Our finding of a 43.2% prevalence of Definite CCAS in this mixed SCA cohort is consistent with findings from a USA SCA natural history cohort (46%)^12^ and with the original CCAS-S development cohort involving various cerebellar conditions (46%).^9^ Our rate of Controls meeting criteria for Definite CCAS (14.0%) was comparable to previous studies (11–22%),^10^  ^,13-15,16^ but higher than that reported in the aforementioned USA SCA natural history cohort and the CCAS-S development cohort (5.4% and 0%, respectively). There continue to be efforts to improve the specificity of the CCAS-S. Reduced rates of Definite CCAS in control groups have been observed after correcting for demographic factors,^31^ and after modifications to task instructions.^32^ This issue also motivates the development of normative data,^33^ given that controls meeting Definite CCAS criteria may reflect normal variability in the population.

Our quantitative between-group comparisons also partially replicated previous reports of population-level reduced CCAS-S performance in SCAs,^12^ specifically our findings of reduced CCAS-S scores in SCA2 and SCA3.^10,11,13,14,16^ We also present new evidence for significantly reduced CCAS-S performance in SCA6, contrary to one previous report whereby reduced performance did not reach statistical significance but group differences trended in the same direction.^14^ However, that previous study included only 14 individuals with SCA6 (relative to *n* = 26 at present); this discrepancy may be due to statistical power. Conversely, while our SCA1 cohort trended towards poorer performance relative to controls, this was not statistically significant, failing to replicate a finding in a cohort of 31 individuals with SCA1 (relative to *n* = 14 here) who had similar average age, onset age, and disease duration characteristics to our cohort.^13^ This discrepancy may similarly relate to differences in statistical power, particularly considering that we observed a moderate effect for higher CCAS-S fail score in the SCA1 group compared to controls.

Our exploratory analysis of group difference effect sizes across CCAS-S domains generated patterns for future investigation. Of interest, a greater deficit in phonemic fluency was observed relative to semantic verbal fluency. This appears in keeping with the executive dysfunction associated with the CCAS-S, where phonemic fluency (naming items beginning with a given letter) involves higher demands on executive processes than does semantic fluency (naming items in a category), due to the role of semantic language networks in facilitating word retrieval in the latter. Furthermore, while there is currently no evidence of distinct deficit profiles for different SCA types across CCAS-S domains, and the differences reported herein should not be considered confirmed findings given the exploratory nature of this analysis, the current results highlight interesting hypotheses for further investigation.

### Education and ataxia severity explain inter-individual variability in CCAS-S performance

We replicated previously-reported associations between poorer CCAS-S performance and higher ataxia motor severity, using patient self-report of motor status on rating scales; however, replication of significant associations with lower education was restricted to our control group..^11,12,14,34^ Overall, we further support ataxia severity as an important cross-sectional correlate of CCAS-S performance. Furthermore, given the finding of small effect sizes for education but negligible effect sizes for age, we add to evidence that age is not a comparably important demographic correlate. The association between education and CCAS-S performance in the Control group indicates that education confers a general advantage. This suggests that educational attainment could confound CCAS designation, and correcting for education may be important in future scale development (note also the demographic-correction formula which has been developed^35^ as described in a study of the German CCAS-S).^15^

The small to moderate relationship between ataxia motor severity and CCAS-S performance is difficult to interpret at the cross-sectional level. These variables may be correlated because they reflect the same underlying level of disease severity and not because they are causally related. Longitudinal studies tracking CCAS-S performance and ataxia motor severity at multiple timepoints would indicate whether this association represents a common progressive course.

### Psychomotor variability is a correlate of CCAS-S performance

Our finding that speed of finger tapping showed moderate to strong covariance with CCAS-S performance further supports the association between CCAS-S and motor measures of ataxia severity.^8^ Amongst other psychomotor tasks posited to have an greater cognitive load, we did not observe associations between task speed and CCAS-S performance; however, we demonstrated significant shared variance between CCAS-S performance and psychomotor *variability*. Performance variability is of interest in SCAs given the cerebellum’s role in maintaining consistency of movement,^2^ mirrored with its role in maintaining consistency of cognitive processes per the Dysmetria of Thought theory.^36^ We demonstrate here greater psychomotor variability associated with poorer cognitive performance. Whether increased psychomotor variability is an index of greater disease severity, which in turn correlates with reduced cognitive performance, or whether psychomotor variability is an underlying factor for reduced cognitive performance due to variability in cognitive processes, is outside the scope of the present work. However, this is a relevant future research question to understand the mechanisms of reduced cognition in SCAs. It is also unclear why variability in some but not other psychomotor tasks was associated with CCAS-S performance.

### CCAS-S performance is related to subjectively-rated cognition

We show for the first time that CCAS-S performance covaries moderately with self-rated cognition for day-to-day activities, but does not correlate to the same extent with more general non-motor symptoms including fatigue, emotional function, and psychosocial function (see next section), supporting the ecological validity of the measure.

Notably, while a moderate, significant correlation was observed against the 7-item PROM-Ataxia Mental 2 (cognitive) subdomain (past 2 weeks), a negligible correlation was observed against the 8-item Neuro-QoL Cognitive Function survey (past 7 days). These scales have largely non-overlapping content, apart from the common inclusion of new learning (Neuro-QoL: ‘*Learning new tasks or instructions’;* PROM-Ataxia: ‘*I can learn new skills’*) and language comprehension (Neuro-QoL: ‘*I had to read something several times to understand it’;* PROM-Ataxia: ‘*I can comprehend the things I have read and/or heard’*). Constructs assessed in the PROM-Ataxia and not in the Neuro-QoL relate to word-finding, recall memory, multitasking, decision-making, and navigation. In contrast, the Neuro-QoL includes items assessing speed of thinking, attention, concentration, reading and following complex instructions, planning, and time management. Our current finding might reflect a better match between the PROM-Ataxia items, developed with input from people with ataxias,^23^ and the CCAS-S. The PROM-Ataxia appears to better capture the cognitive features of the CCAS (linguistic, visuospatial, and executive function) than the Neuro-QoL, which does not contain a visuospatial item. A study limitation is that control participants completed the Neuro-QoL but not the PROM-Ataxia scale, therefore we cannot compare the scales’ abilities to show group differences. Regardless, our results suggest that the set of cognitive functions selected in research studies may influence the relationships that can be identified between cognition and other features of SCAs. It also indicates that CCAS-S performance may not necessarily relate to general cognition, but specific aspects thereof. This preliminary evidence motivates further exploration, including qualitative approaches, into the range of cognitive functions impacted amongst individuals with SCAs.

### Minimal contribution of emotional function and fatigue to CCAS-S performance

We report small associations between depression symptoms and CCAS-S performance, at trending statistical significance for total fail score. Small effect sizes for this association were also observed in a USA cohort using Patient Health Questionnaire-9^37^ scores amongst 93 individuals with SCA3 and 113 individuals with SCA1, 2, 6, 7, and 8.^12^ We did not observe correlations between self-reported fatigue and CCAS-S performance (*r* < 0.06), in contrast to small to medium correlations reported amongst 105 individuals with SCA3 and a combined SCA group (*n* = 116) in the USA cohort, using the Fatigue Severity Scale^38^ as the outcome measure. This was the first study to examine associations between CCAS-S performance and emotional dysregulation or psychosocial function, with negligible to small, non-significant associations observed.

Taken together, these results indicate that emotional function, psychosocial function, and fatigue do not appear to represent key risk factors for cognitive impairment in SCAs 1, 2, 3 and 6. Taken another way, it does not appear that depression and fatigue, features of SCAs also known to impact cognitive performance, explain the presence of cognitive deficits in this population.

### Limitations and future directions

Our online administration of the CCAS-S may not be directly comparable to previous in-person assessments; differences between in-person and remote video-based CCAS-S outcomes have been reported.^16^ This may limit the generalisability of our findings to the wider, growing literature on CCAS-S outcomes. We also acknowledge that while our remote assessment methodology provided increased access to research participation, participant outcomes may have been impacted by inconsistent testing environments and different levels of familiarity with digital devices. Given that all our participants were tested remotely (including healthy controls), these factors likely did not impact on our inferences regarding group differences, but they may have introduced additional noise into the data. Furthermore, the requirements of our study design may have also excluded individuals without access to or support for using the required digital technology, introducing bias in our sampling.

Furthermore, we did not have scope within our remote design to conduct standard clinician-rated assessments of motor ataxia symptoms such as the SARA or the Brief Ataxia Rating Scale (BARS). We instead relied on participant self-report of motor function as captured via structured questionnaires (PROM-Ataxia Physical domain, Functional Staging for Ataxia scale). However, we note that the Physical domain of the PROM-Ataxia scale is strongly correlated with the SARA in SCAs, as observed in the Clinical Research Consortium for Spinocerebellar Ataxia (*r*_s_ = 0.70, *P* < 0.05)^39^ and in a Chinese cohort (*r* = 0.809, *P* < 0.001).^40^ As such, the correlations reported here likely reflect the correlations that would be observed against the SARA and similar scales.

We also acknowledge that due to absence of SARA score, we were not able to apply a SARA-based criterion to define symptomatic participants, as applied in previous studies (i.e. SARA ≥3).^41,42^

Furthermore, while all participants were fluent English speakers, we did not collect information about whether English was their first language or a subsequent language. English language status could have an impact on the language-based cognitive tasks applied in this study.

We also relied on self-reported cognitive and emotional function. Reduced insight can be a feature of cognitive and neuropsychiatric conditions, including as part of a dysexecutive syndrome.^43^ Reduced insight in some participants could potentially attenuate inter-individual variability in self-reported cognition and impact the ability to evaluate its relationship to CCAS-S performance. It would be useful to investigate, for example, whether an informant report of cognition has a stronger association with objectively-measured cognition, compared to self-report.

We also note that the Affective component of the CCAS is measured in the CCAS-S by a symptom checklist contributing 6 points to the scale. Further work investigating the correlates of this component of the CCAS should include use of the recently-published Cerebellar Neuropsychiatric Rating Scale (CNRS)^44^ which provides a more detailed assessment of affective/neuropsychiatric symptoms and their severity. We also acknowledge that while depression and anxiety are commonly reported in people with SCA,^21^ it is currently not possible to disambiguate psychiatric symptoms directly related to the cerebellar pathology from independent, comorbid mental health conditions. Furthermore, future work should include a motor speech task to better understand the influence of dysarthria on CCAS-S performance, as has been reported in other work.^33,45,46^

We acknowledge that many correlations were undertaken, and some of the significant correlations would not survive correction for multiple comparisons. Although it is valuable to investigate a broad range of potential correlates of CCAS-S performance in an exploratory fashion in the first instance, it will be necessary to confirm these relationships in an *a priori* manner in future work.

Finally, the present results do not provide insights into the neuroanatomical basis of cognitive deficits in SCAs. While the CCAS can explain cognitive deficits arising from the cerebellar degeneration seen in SCAs, aberrations are also reported in the cerebrum and basal ganglia^1,47^ and could also contribute to or explain cognitive deficits in this cohort. CCAS-S performance was linked to cerebellar imaging features in a chronic stoke cohort^48^; it would be informative to explore imaging correlates of CCAS-S performance in SCAs. A potential motivation for this approach would be to understand whether imaging features provide insights into predicting risk for CCAS in SCA.

In conclusion, this study provides convergent evidence for impaired cognition in SCAs as indexed by the CCAS-S, and shows that performance on this measure is related to motor features of the disease, psychomotor variability, and, importantly, subjectively-reported day-to-day cognitive functioning. This work motivates further investigation into the patterns, progression, and correlates of cognition in this cohort. Key areas for future research are understanding the role of psychomotor variability in cognitive performance, and identifying which aspects of everyday cognition best capture the manifestation of cognitive difficulties.

## Supplementary Material

fcaf425_Supplementary_Data

## Data Availability

The data that support the findings of this study are available from the corresponding author, upon reasonable request. The R code used for the analysis and preparation of figures is available at the following URL: https://doi.org/10.26180/30229738
