# Supplementary material for: Psychomotor and non-motor correlates of cognition in spinocerebellar ataxias Types 1, 2, 3, and 6
Source: Brain Commun. 2025 Oct 28;7(6):fcaf425. doi: 10.1093/braincomms/fcaf425 (PMC12646072; doi:10.1093/braincomms/fcaf425)
Supplement: fcaf425_Supplementary_Data [file fcaf425_supplementary_data.docx]

**Psychomotor and non-motor correlates of cognition in spinocerebellar ataxias types 1, 2, 3, and 6**

Louisa P. Selvadurai, Sheryl Gullia, James Morgan, Sarah J. Wallis, Kishore R. Kumar, David J. Szmulewicz, Ian H. Harding

**SUPPLEMENTARY MATERIALS**

**Caption:** Additional methodological and statistical information.

**Supplementary Table 1:** Statistical information for demographic matching between SCA groups and matched Control groups.

|  | **SCA M(SD)** | **CON** | **Test** | **Statistic** | ***p-value*** |
| --- | --- | --- | --- | --- | --- |
| **SCA1** | *n* = 14 | *n* = 14 |  |  |  |
| age | 45.14(11.63) | 47.86(15.93) | t-test | 0.515 | 0.611 |
| sex | 10 females | 9 females | chi-square | 0.000 | 1.000 |
| education | 16.43(2.95) | 16.14(4.04) | t-test | -0.214 | 0.833 |
| **SCA2** | *n* = 16 | *n* = 16 |  |  |  |
| age | 55.12(12.63) | 55.12(13.99) | t-test | 0.000 | 1.000 |
| sex | 9 females | 10 females | chi-square | 0.000 | 1.000 |
| education | 17.69(2.75) | 18.31(2.7) | t-test | 0.649 | 0.522 |
| **SCA3** | *n* = 18 | *n* = 18 |  |  |  |
| age | 46.5(11.8) | 47.28(12.05) | t-test | 0.196 | 0.846 |
| sex | 11 females | 10 females | chi-square | 0.000 | 1.000 |
| education | 16.94(2.96) | 17.33(3.38) | t-test | 0.367 | 0.716 |
| **SCA6** | *n* = 26 | *n* = 26 |  |  |  |
| age | 62.12(11.46) | 60.73(12.09) | t-test | -0.424 | 0.673 |
| sex | 13 females | 15 females | chi-square | 0.077 | 0.781 |
| education | 14.5(4.58) | 15.27(3.31) | t-test | 0.694 | 0.491 |

**Supplementary Table 2.** Adaptation of CCAS-S for Cerebellar Cognitive Affective Syndrome Scale (CCAS-S) for teleconference-mediated administration.

| **CCAS-S domain** | **Original administration** | **Teleconference-adapted administration** |
| --- | --- | --- |
| Cube draw/copy | Examinee draws cube on CCAS-S record form in response to verbal instruction.  If copying is required due to inaccurate drawing, examinee views the image of a cube on the record form and copies it on the form. | Examinee draws cube on piece of paper in response to verbal instruction, and shows drawing to webcam. The examiner takes a screenshot of the drawing in real time, for scoring.  If copying is required due to inaccurate drawing, examiner presents a digital image of a cube on the computer screen via screensharing. Examinee copies the cube on a piece of paper and shows drawing to webcam.  The examiner takes a screenshot of the drawing in real time, for scoring. |
| Go no-go | Examiner performs a series of single or double taps on the table. Examinee raises finger when the examiner taps once, and does nothing when the examiner taps twice. | Examiner engages in alternative tapping activity with hand raised in view of webcam (e.g., tapping between thumb and other fingers, tapping fist on a raised surface).  Examinee raises hand in view of webcam when the examiner taps once, and does nothing when the examiner taps twice. |


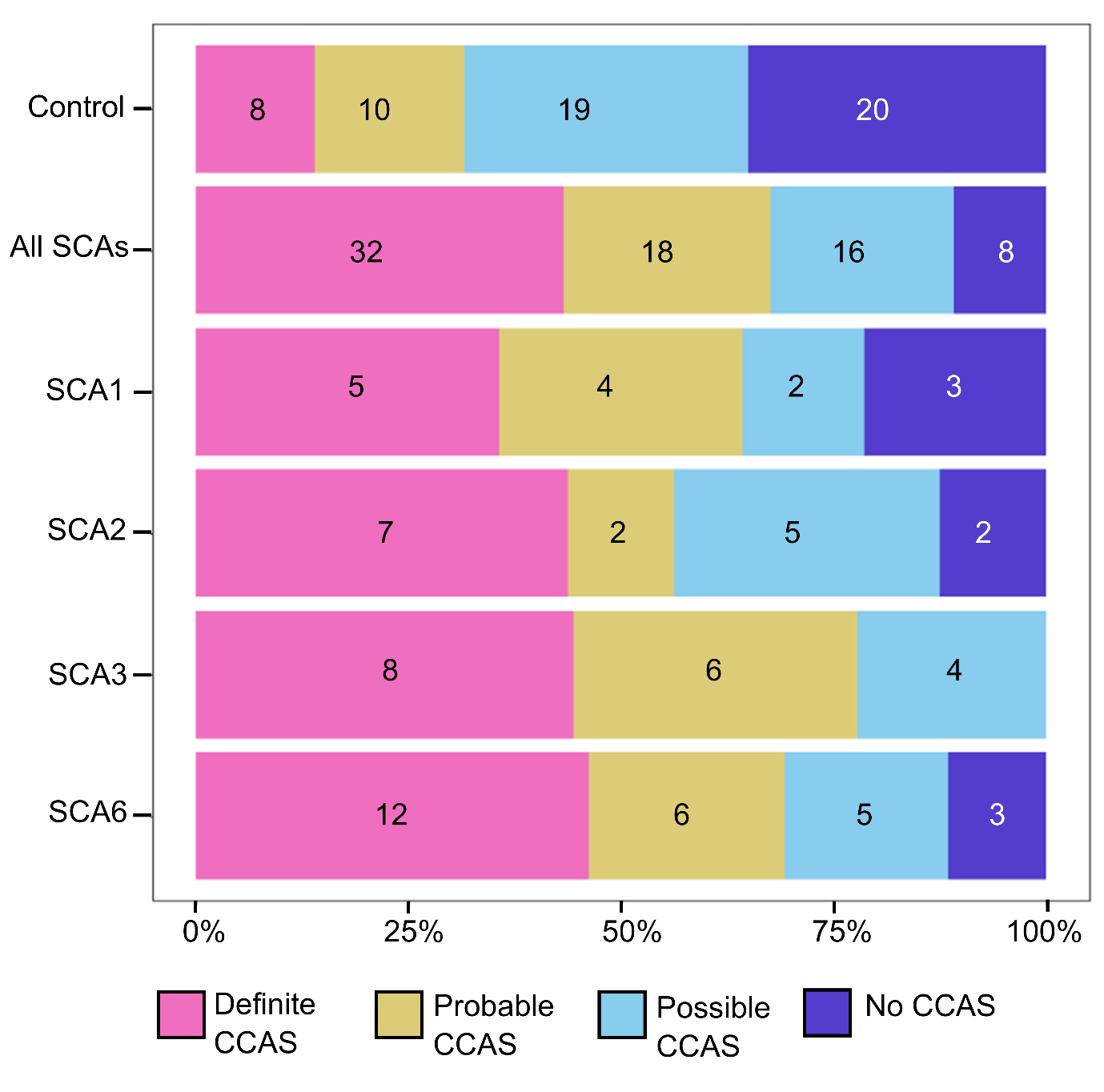


**Supplementary Figure 1**: **CCAS-S category designation by group and SCA type.** Horizontal stacked bar chart showing the proportion (%) of individuals meeting criteria for Definite, Probable, Possible, and No CCAS, within the following groups: Control, All SCAs, SCA1, SCA2, SCA3, and SCA6. The raw number of individuals in each category is overlaid on each section of the relevant bar. SCA = spinocerebellar ataxia.

**Supplementary Tables 3-6:** Descriptive statistics for CCAS-S performance (total raw, total fail, domain scores) by group, and inferential statistics for group comparisons. Mann-Whitney *U*-tests used for all comparisons.

**Supplementary Table 3.** SCA1 vs. matched control group (CON1)

|  | **SCA1, *n* = 14** | **CON1, *n* = 14** | **Statistic** | **Effect size** | ***p*-value** |
| --- | --- | --- | --- | --- | --- |
| Total raw | 93.71(11.33), [74-111], n=14 | 99.14(14.43), [66-116], n=14 | 125 | 0.276 | 0.223 |
| Total fails | 1.93(1.38), [0-4], n=14 | 1.21(1.48), [0-5], n=14 | 66.5 | -0.321 | 0.143 |
| Semantic fluency | 18.43(4.5), [11-26], n=14 | 20.71(4.48), [12-26], n=14 | 127.5 | 0.301 | 0.18 |
| Phonemic fluency | 12.71(4.38), [3-18], n=14 | 14.07(4.6), [6-19], n=14 | 118.5 | 0.209 | 0.356 |
| Category switching | 10.79(3.72), [3-15], n=14 | 12.64(2.5), [7-15], n=14 | 128.5 | 0.311 | 0.163 |
| Digit span forward | 6.36(1.22), [4-8], n=14 | 6.71(0.91), [5-8], n=14 | 111.5 | 0.138 | 0.533 |
| Digit span backward | 4.36(0.63), [3-5], n=14 | 4.57(0.94), [3-6], n=14 | 107 | 0.092 | 0.67 |
| Cube drawing | 13.57(1.74), [11-15], n=14 | 13.93(1.9), [9-15], n=14 | 112 | 0.143 | 0.465 |
| Verbal recall | 13.14(2.74), [5-15], n=14 | 12.14(2.85), [6-15], n=14 | 73.5 | -0.25 | 0.249 |
| Similarities | 7.86(0.36), [7-8], n=14 | 7.86(0.36), [7-8], n=14 | 98 | 0 | 1 |
| Go No-Go | 1.43(0.85), [0-2], n=14 | 1.86(0.36), [1-2], n=14 | 122 | 0.245 | 0.154 |
| Affect | 5.07(1.21), [3-6], n=14 | 4.64(1.91), [1-6], n=14 | 90 | -0.082 | 0.707 |

***Note:*** SCA/CON descriptive statistics: M(SD), [range], group size. Effect size = point biserial *r*

**Supplementary Table 4.** SCA2 vs. matched control group (CON2)

|  | **SCA2, *n* = 16** | **CON2, *n* = 16** | **Statistic** | **Effect size** | ***p*-value** |
| --- | --- | --- | --- | --- | --- |
| Total raw | 94(11.27), [73-112], n=16 | 103.56(9.87), [85-117], n=16 | 190 | 0.484 | **0.020** |
| Total fails | 2.12(1.5), [0-5], n=16 | 1(1.26), [0-4], n=16 | 70 | -0.453 | **0.025** |
| Semantic fluency | 20.88(3.86), [14-26], n=16 | 22(4.7), [11-26], n=16 | 158 | 0.234 | 0.260 |
| Phonemic fluency | 10.44(4.59), [2-19], n=16 | 15.06(2.98), [10-19], n=16 | 206 | 0.609 | **0.003** |
| Category switching | 12.12(2.6), [7-15], n=16 | 13(2.16), [8-15], n=16 | 154.5 | 0.207 | 0.319 |
| Digit span forward | 5.88(1.02), [4-8], n=16 | 6.62(1.31), [5-8], n=16 | 169 | 0.32 | 0.115 |
| Digit span backward | 4.44(1.15), [3-6], n=16 | 4.94(1.12), [3-6], n=16 | 158.5 | 0.238 | 0.231 |
| Cube drawing | 13.06(1.91), [9-15], n=16 | 14.44(1.21), [12-15], n=16 | 179 | 0.398 | **0.025** |
| Verbal recall | 12.94(2.02), [9-15], n=16 | 12.06(2.89), [6-15], n=16 | 110 | -0.141 | 0.501 |
| Similarities | 7.62(0.72), [6-8], n=16 | 7.88(0.34), [7-8], n=16 | 146 | 0.141 | 0.332 |
| Go No-Go | 1.5(0.73), [0-2], n=16 | 1.94(0.25), [1-2], n=16 | 169 | 0.32 | **0.034** |
| Affect | 5.12(1.41), [1-6], n=16 | 5.62(0.89), [3-6], n=16 | 152.5 | 0.191 | 0.253 |

***Note:*** SCA/CON descriptive statistics: M(SD), [range], group size. Effect size = point biserial *r*

**Supplementary Table 5.** SCA3 vs. matched control group (CON3)

|  | **SCA3, *n* = 18** | **CON3, *n* = 18** | **Statistic** | **Effect size** | ***p*-value** |
| --- | --- | --- | --- | --- | --- |
| Total raw | 85.94(12.68), [55-103], n=18 | 100.28(11.13), [79-116], n=18 | 262 | 0.617 | **0.002** |
| Total fails | 2.83(1.65), [1-6], n=18 | 1.5(1.2), [0-4], n=18 | 87.5 | -0.46 | **0.016** |
| Semantic fluency | 16.78(6.41), [5-26], n=18 | 21.56(3.84), [15-26], n=18 | 238.5 | 0.472 | **0.015** |
| Phonemic fluency | 7.61(3.2), [1-12], n=18 | 15.17(3.57), [8-19], n=18 | 301 | 0.858 | **< .001** |
| Category switching | 9.83(3.09), [5-13], n=18 | 11.44(2.73), [6-15], n=18 | 205 | 0.265 | 0.176 |
| Digit span forward | 6.06(1.06), [4-8], n=18 | 6.28(1.02), [5-8], n=18 | 177 | 0.093 | 0.632 |
| Digit span backward | 4.28(0.96), [3-6], n=18 | 4.17(1.29), [1-6], n=18 | 158 | -0.025 | 0.908 |
| Cube drawing | 14.28(1.41), [11-15], n=18 | 13.89(1.97), [9-15], n=18 | 150.5 | -0.071 | 0.646 |
| Verbal recall | 13.83(1.86), [9-15], n=18 | 13.11(2.45), [8-15], n=18 | 132.5 | -0.182 | 0.319 |
| Similarities | 7.44(0.98), [4-8], n=18 | 7.78(0.43), [7-8], n=18 | 191 | 0.179 | 0.261 |
| Go No-Go | 1.83(0.38), [1-2], n=18 | 1.83(0.38), [1-2], n=18 | 162 | 0 | 1 |
| Affect | 4(1.81), [1-6], n=18 | 5.06(1.59), [1-6], n=18 | 218.5 | 0.349 | 0.057 |

***Note:*** SCA/CON descriptive statistics: M(SD), [range], group size. Effect size = point biserial *r*

**Supplementary Table 6.** SCA6 vs. matched control group (CON6)

|  | **SCA6, *n* = 26** | **CON6, *n* = 26** | **Statistic** | **Effect size** | ***p*-value** |
| --- | --- | --- | --- | --- | --- |
| Total raw | 90.5(15.34), [54-115], n=26 | 100.04(11.57), [66-117], n=26 | 474.5 | 0.404 | **0.013** |
| Total fails | 2.85(2.36), [0-9], n=26 | 1.31(1.19), [0-5], n=26 | 192 | -0.432 | **0.006** |
| Semantic fluency | 19.65(5.61), [11-26], n=26 | 22.58(4.01), [12-26], n=26 | 443 | 0.311 | 0.051 |
| Phonemic fluency | 10.27(5.16), [0-19], n=26 | 14(4.11), [6-19], n=26 | 481.5 | 0.425 | **0.009** |
| Category switching | 12.15(2.69), [7-15], n=26 | 12.54(2.61), [6-15], n=26 | 366.5 | 0.084 | 0.601 |
| Digit span forward | 5.77(1.18), [4-8], n=26 | 6.35(0.89), [4-8], n=26 | 442.5 | 0.309 | **0.047** |
| Digit span backward | 4(1.13), [2-6], n=26 | 4.15(1.12), [1-6], n=26 | 368 | 0.089 | 0.575 |
| Cube drawing | 13.19(2.84), [5-15], n=26 | 13.69(1.78), [9-15], n=26 | 350 | 0.036 | 0.808 |
| Verbal recall | 11.69(2.66), [6-15], n=26 | 12.27(2.27), [6-15], n=26 | 379.5 | 0.123 | 0.449 |
| Similarities | 6.96(1.18), [3-8], n=26 | 7.81(0.4), [7-8], n=26 | 490.5 | 0.451 | **0.001** |
| Go No-Go | 1.31(0.93), [0-2], n=26 | 1.81(0.4), [1-2], n=26 | 423 | 0.251 | 0.052 |
| Affect | 5.5(1.36), [1-6], n=26 | 4.85(1.87), [0-6], n=26 | 268.5 | -0.206 | 0.114 |

***Note:*** SCA/CON descriptive statistics: M(SD), [range], group size. Effect size = point biserial *r*


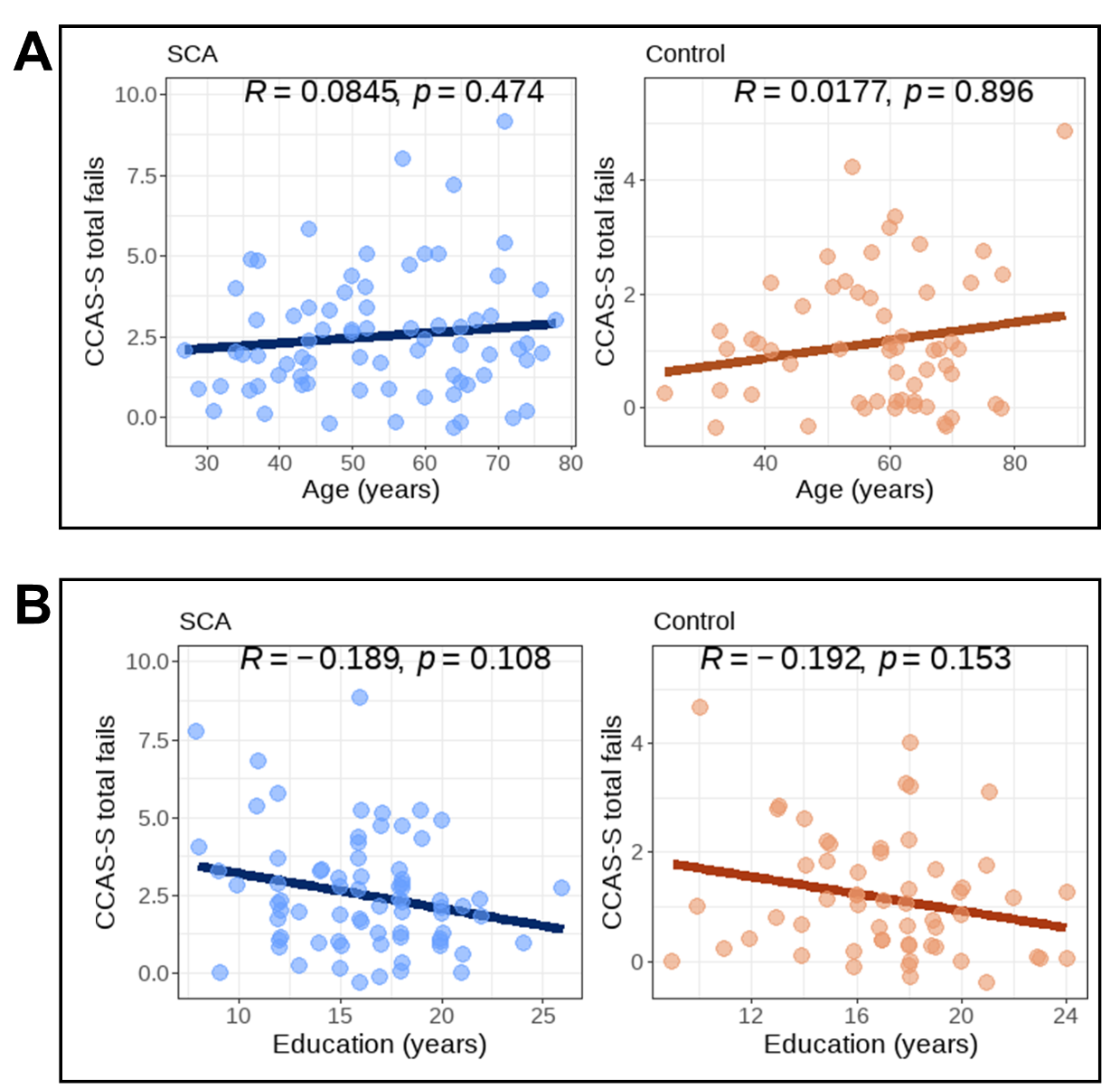


**Supplementary Figure 2. CCAS-S total fails vs. demographic variables.** Scatterplots showing the relationship between CCAS-S number of failed items and A) age and B) years of education in the combined SCA (*n* = 74) and Control (*n* = 57) groups. Each datapoint represents a single participant. *R* = Spearman correlation coefficient. Asterisk indicates a significant correlation at *p* < .05. CCAS-S = Cerebellar Cognitive Affective Syndrome Scale; SCA = spinocerebellar ataxia.


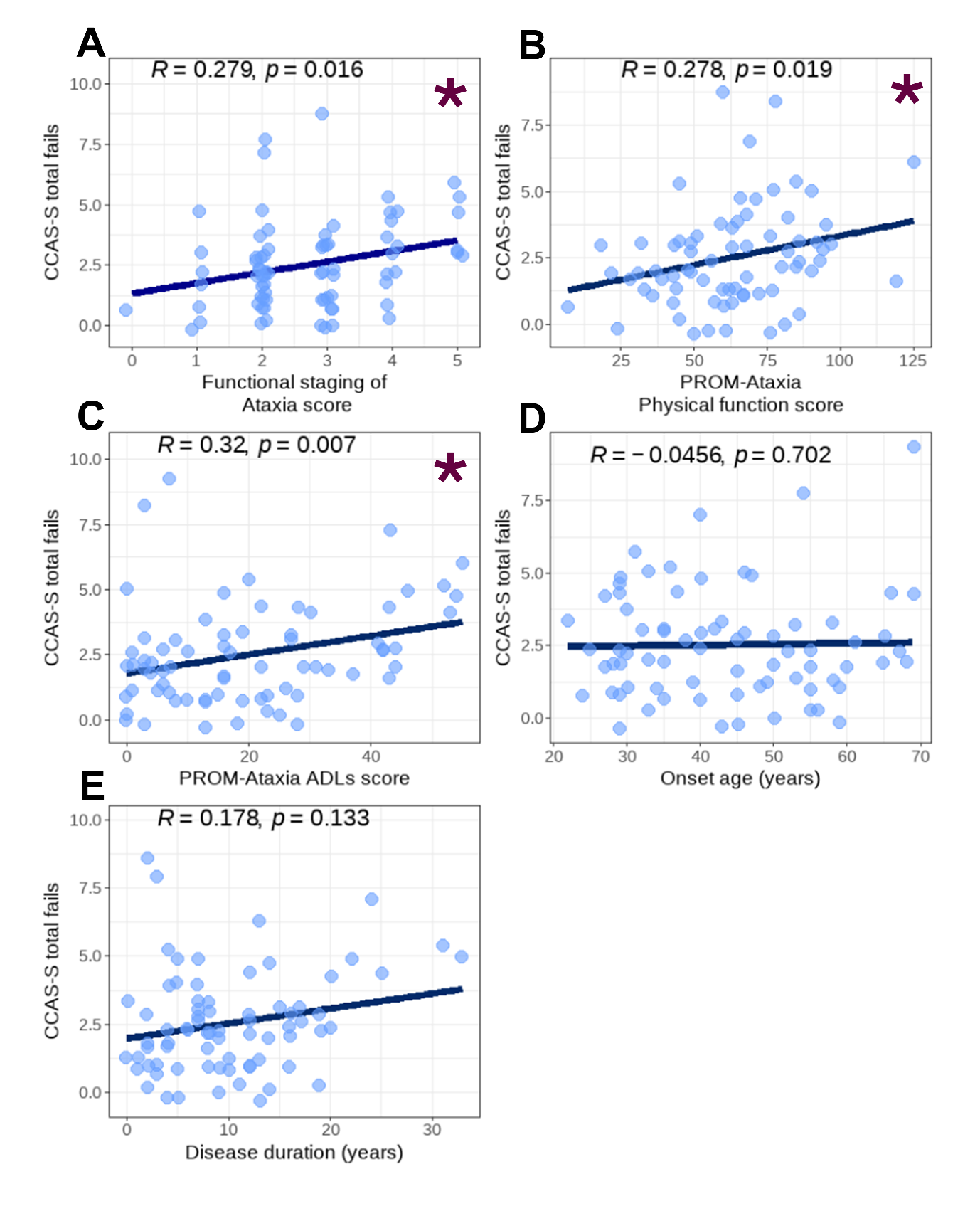


**Supplementary Figure 3. CCAS-S total fails vs. clinical variables.** Scatterplots showing the relationship between CCAS-S number of failed items and clinical variables: (A) Functional Staging of Ataxia score (*n* = 74); PROM-Ataxia (B) Physical function and (C) ADLs (*n* = 71), (D) onset age (*n* = 73) and (E) disease duration (*n* = 73). Each datapoint represents a single participant. *R* = Spearman correlation coefficient. Asterisk indicates a significant correlation at *p* < .05. CCAS-S = Cerebellar Cognitive Affective Syndrome Scale; PROM-Ataxia = Patient-Reported Outcome Measure of Ataxia; ADLs = Activities of Daily Living.

**
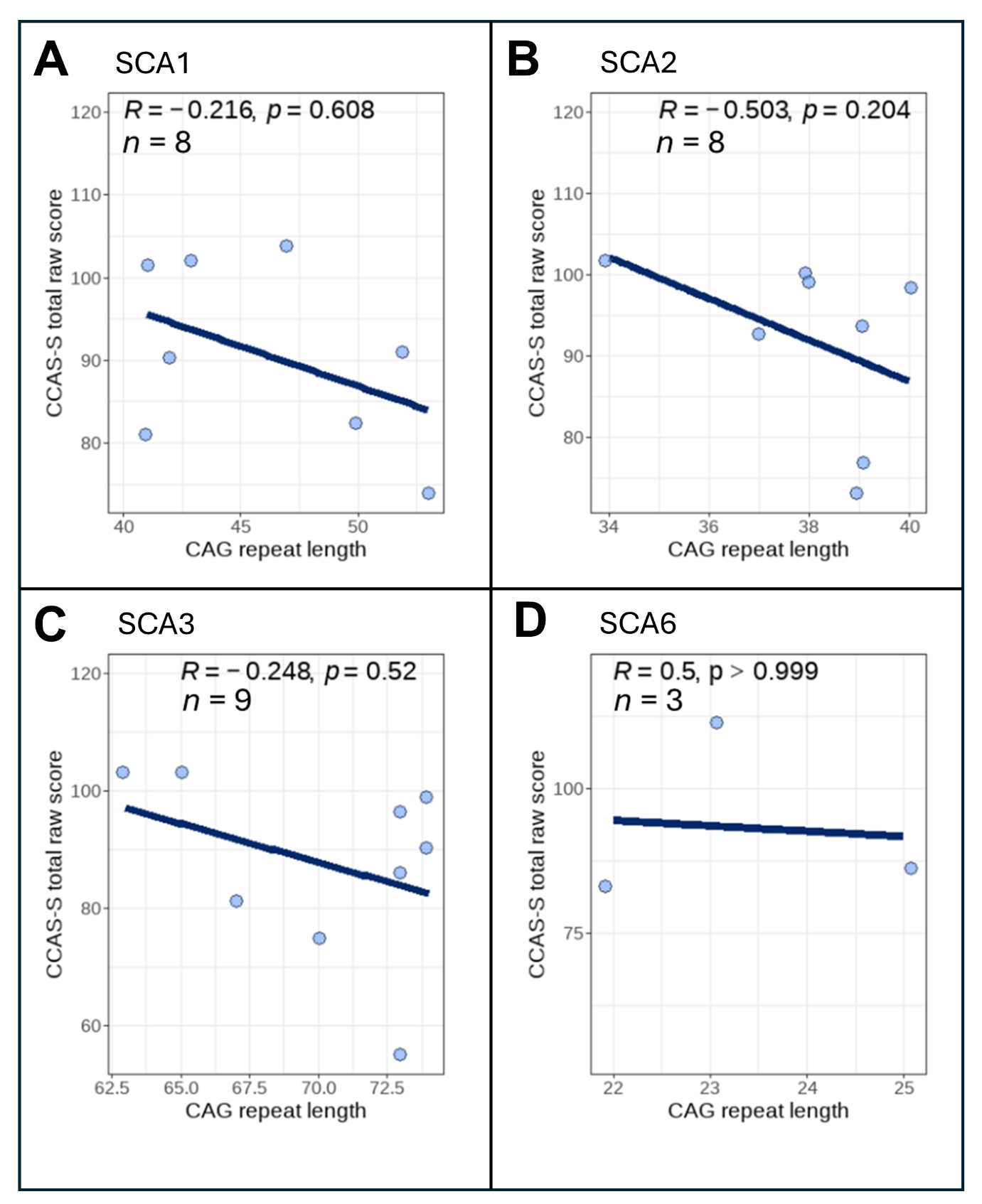
**

**Supplementary Figure 4. CCAS-S raw score vs. CAG repeat length by SCA type.** Scatterplots for each of A) SCA1 (*n* = 8), B) SCA2 (*n* = 8), C) SCA3 (*n* = 9), and D) SCA6 (*n* = 3), showing the relationship between CCAS-S total raw score and CAG repeat length. Each datapoint represents a single participant. *R* = Spearman correlation coefficient. CCAS-S = Cerebellar Cognitive Affective Syndrome Scale.


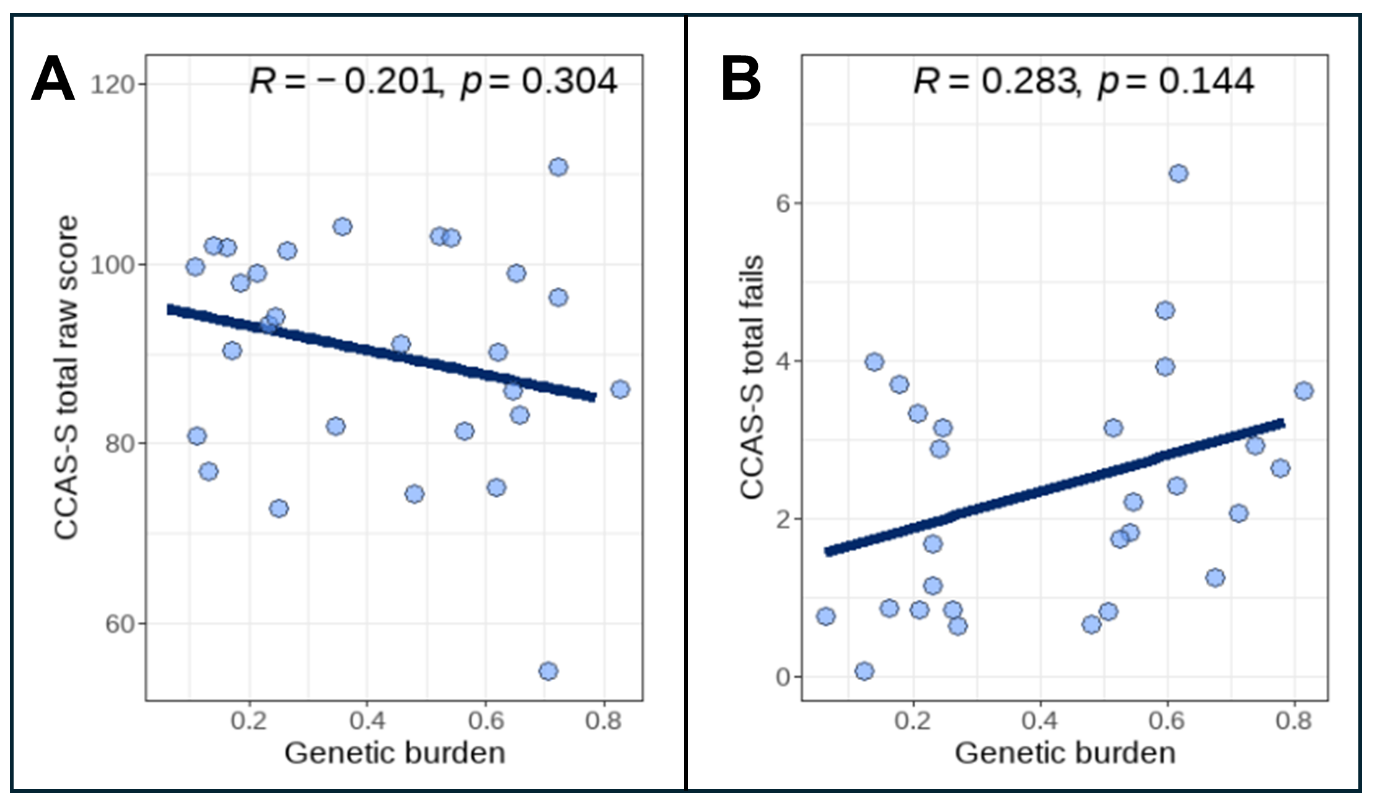


**Supplementary Figure 5. CCAS-S performance vs. genetic burden score CAG repeat length.** Scatterplots showing the relationship between A) CCAS-S total raw score and B) CCAS-S number of failed items vs. genetic burden value. Within each SCA genotype, genetic burden was calculated as: (CAG repeat length – maximum healthy repeat length)/Maximum healthy repeat length. Maximum healthy repeat length was defined as follows: SCA1 = 35; SCA2 = 32; SCA3 = 44; SCA6 = 14. Each datapoint represents a single participant. *R* = Spearman correlation coefficient. CCAS-S = Cerebellar Cognitive Affective Syndrome Scale.

**Supplementary Table 7.** Descriptive statistics for psychomotor task outcomes (SCA group)

| **Variable** | **N** | **Median** | **Min** | **Max** |
| --- | --- | --- | --- | --- |
| Speeded tapping - median | 51 | 251 | 149 | 509 |
| Paced tapping – median | 61 | 522.5 | 239.5 | 666 |
| Visual reaction time – median | 65 | 353.5 | 211.5 | 674.5 |
| Decision time – median | 63 | 266 | -48 | 1518.5 |
| Inhibition time – median | 65 | 28.5 | -832 | 232.5 |
|  |  |  |  |  |
| Speeded tapping - MAD | 51 | 28.91 | 13.34 | 140.85 |
| Paced tapping – MAD | 61 | 63.01 | 19.27 | 327.65 |
| Visual reaction time – MAD | 65 | 45.22 | 13.34 | 214.24 |
| Decision time – MAD | 63 | 51.15 | -40.03 | 1376.59 |
| Inhibition time – MAD | 65 | -1.48 | -1189.79 | 269.09 |

***Note:*** MAD = median absolute deviation

**Supplementary Table 8.** Descriptive statistics for subjective non-motor measures (SCA group)

| **Variable** | **Range** | **N** | **Mean** | **SD** | **Min** | **Max** |
| --- | --- | --- | --- | --- | --- | --- |
| Neuro-QoL Cognitive Function | 8-40 | 74 | 32.64 | 6.57 | 13 | 40 |
| Neuro-QoL Depression | 8-40 | 74 | 13.27 | 5.9 | 8 | 32 |
| Neuro-QoL Emotional and Behavioral Dyscontrol | 8-40 | 74 | 15.34 | 6.77 | 8 | 37 |
| Neuro-QoL Fatigue | 8-40 | 74 | 19.55 | 7.87 | 8 | 37 |
| PROM-Ataxia psychosocial (Mental 1) | 0-40 | 71 | 13.58 | 7.97 | 0 | 32 |
| PROM-Ataxia cognitive (Mental 2) | 0-28 | 71 | 6.7 | 5.43 | 0 | 23 |


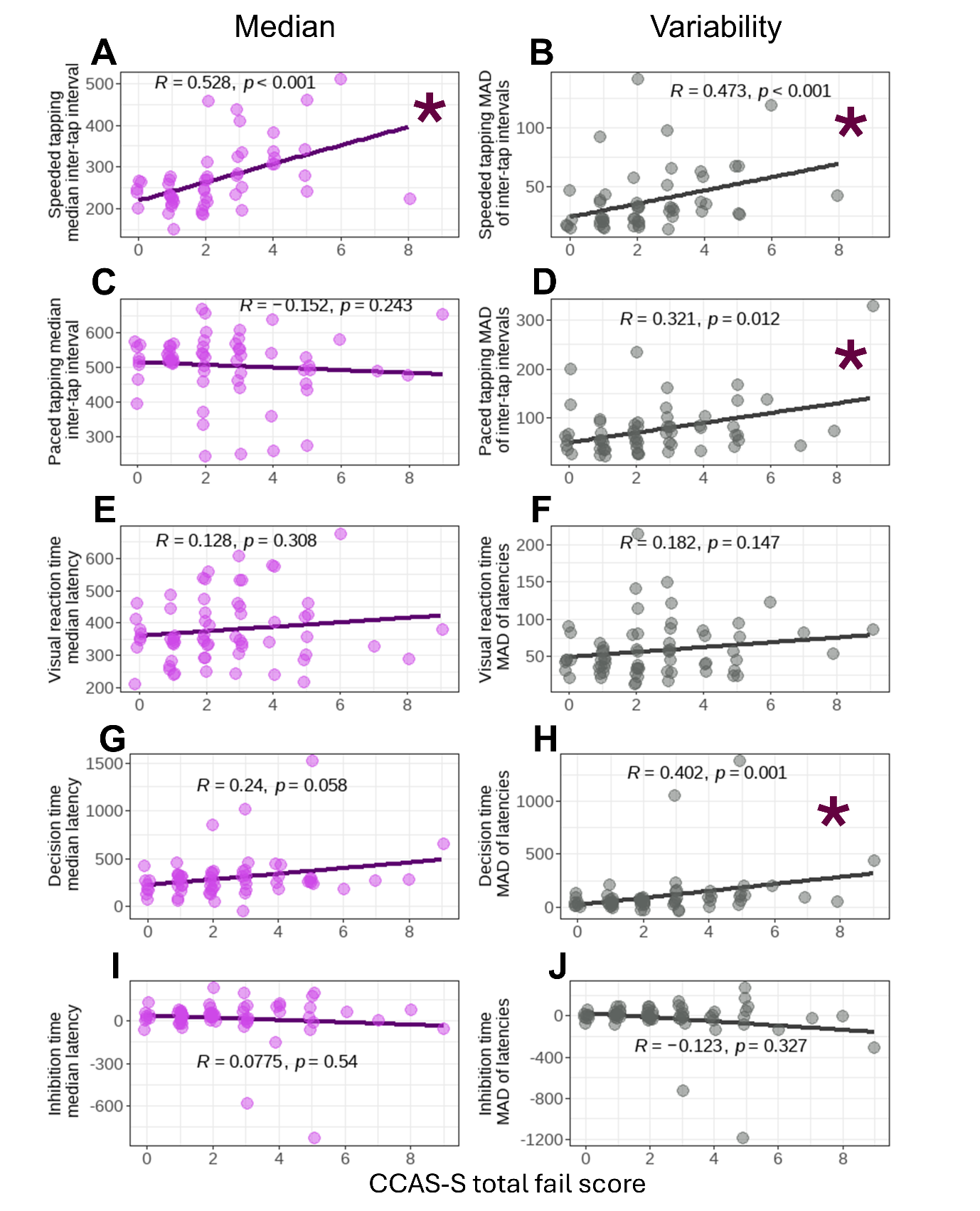


**Supplementary Figure 6. CCAS-S total fails vs. psychomotor variables.** Scatterplots showing the relationship between CCAS-S total fails and median (left column) and median absolute deviation (MAD; right column) performance on psychomotor tasks: (A-B) speeded tapping (*n*=51), (C-D) paced tapping (*n*=61), (E-F) visual reaction time (*n*=65), (G-H) decision time (*n*=63) and (I-J) inhibition time (*n*=65). Each datapoint represents a single participant. *R* = Spearman correlation coefficient. Asterisk indicates a significant correlation at *p* < .05. CCAS-S = Cerebellar Cognitive Affective Syndrome Scale; MAD = median absolute deviation; measure of variability.


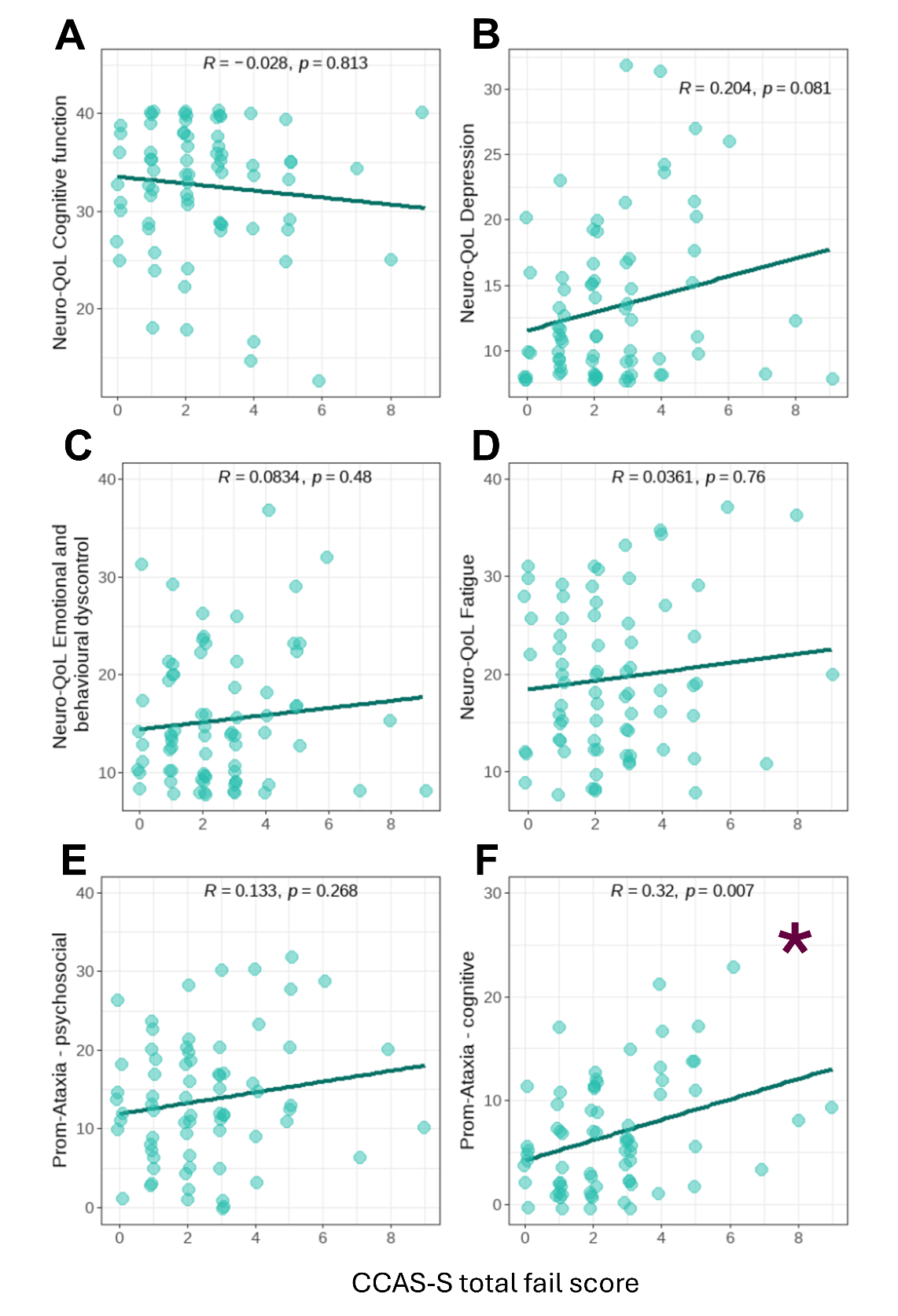


**Supplementary Figure 7. CCAS-S total fails vs. subjective non-motor variables.** Scatterplots showing the relationship between CCAS-S total fails and subjective non-motor outcomes; NeuroQoL scales (*n*=74) – (A) Cognitive function, (B) Depression, (C) Emotional and behavioural dyscontrol, (D) Fatigue; PROM-Ataxia (*n*=71) – (E) Mental 1 (psychosocial) and (F) Mental 2 (cognitive). Each datapoint represents a single participant. *R* = Spearman correlation coefficient. Asterisk indicates a significant correlation at *p* < .05. CCAS-S = Cerebellar Cognitive Affective Syndrome Scale; NeuroQoL = Quality of Life in Neurological Disorders; PROM-Ataxia = Patient-Reported Outcome Measure of Ataxia.

**Supplementary Table 9.** Multiple regression results

| **Formula = CCAS verbal fluency combined score ~ PROM-Ataxia Physical score + PROM-Ataxia Mental 2 score** | | | | |
| --- | --- | --- | --- | --- |
|  |  |  |  |  |
| **Residuals:** |  |  |  |  |
| **Min** | **IQ** | **Median** | **3Q** | **Max** |
| -19.482 | -6.144 | 0.875 | 5.709 | 18.304 |
|  |  |  |  |  |
| **Coefficients:** |  |  |  |  |
|  | **Estimate** | **Standard Error** | ***t*-value** | ***p*-value** |
| (Intercept) | 47.896 | 3.519 | 13.611 | <2e-16*** |
| PROM-Ataxia Physical | -0.06424 | 0.06545 | -0.981 | 0.330 |
| PROM-Ataxia Mental 2 | -0.50440 | 0.27673 | -1.823 | 0.073 |
|  |  |  |  |  |
| Multiple R-squared: 0.132; Adjusted R-squared: 0.107 | | | | |
| F(2, 68) = 5.19, *p* = .008 | | | | |

*** = *p* < .001

**Supplementary Table 10.** Correlations between CCAS-S performance and demographic, clinical, psychomotor, and non-motor variables within each SCA type group

|  | **SCA1** | | **SCA2** | | **SCA3** | | **SCA6** | |
| --- | --- | --- | --- | --- | --- | --- | --- | --- |
|  | CCAS-S raw | CCAS-S fails | CCAS-S raw | CCAS-S fails | CCAS-S raw | CCAS-S fails | CCAS-S raw | CCAS-S fails |
| Age | n=14, r=-0.08, p=0.79 | n=14, r=0.28, p=0.33 | n=16, r=-0.23, p=0.4 | n=16, r=0.15, p=0.59 | n=18, r=-0.04, p=0.86 | n=18, r=0.11, p=0.67 | n=26, r=0.13, p=0.53 | n=26, r=-0.23, p=0.26 |
| Education (years) | n=14, r=0.06, p=0.83 | n=14, r=0.05, p=0.88 | n=16, r=0.26, p=0.33 | n=16, r=-0.41, p=0.11 | n=18, r=0.43, p=0.08 | n=18, r=-0.37, p=0.13 | n=26, r=0.27, p=0.19 | n=26, r=-0.24, p=0.23 |
| Functional Staging of Ataxia | n=14, r=-0.1, p=0.74 | n=14, r=0.31, p=0.29 | n=16, r=-0.31, p=0.24 | n=16, r=0.37, p=0.16 | n=18, r=-0.42, p=0.09 | n=18, r=0.62, p=0.01 | n=26, r=-0.08, p=0.69 | n=26, r=-0.11, p=0.59 |
| PROM-Ataxia Physical | n=14, r=-0.17, p=0.56 | n=14, r=0.1, p=0.74 | n=15, r=-0.42, p=0.12 | n=15, r=0.62, p=0.01 | n=17, r=-0.39, p=0.13 | n=17, r=0.45, p=0.07 | n=25, r=-0.27, p=0.2 | n=25, r=0.13, p=0.53 |
| Onset age | n=14, r=-0.04, p=0.89 | n=14, r=0.22, p=0.45 | n=15, r=0.12, p=0.68 | n=15, r=-0.29, p=0.29 | n=18, r=-0.25, p=0.31 | n=18, r=0.14, p=0.58 | n=26, r=0.08, p=0.68 | n=26, r=-0.13, p=0.54 |
| Duration | n=14, r=-0.29, p=0.31 | n=14, r=0.29, p=0.31 | n=15, r=-0.68, p=0 | n=15, r=0.85, p=0 | n=18, r=0.21, p=0.41 | n=18, r=0, p=0.99 | n=26, r=-0.07, p=0.73 | n=26, r=0.03, p=0.89 |
| Speeded tapping - median | n=11, r=-0.48, p=0.14 | n=11, r=0.66, p=0.03 | n=12, r=-0.18, p=0.57 | n=12, r=0.57, p=0.05 | n=14, r=-0.54, p=0.05 | n=14, r=0.66, p=0.01 | n=14, r=-0.48, p=0.08 | n=14, r=0.37, p=0.19 |
| Speeded tapping - MAD | n=11, r=-0.47, p=0.15 | n=11, r=0.63, p=0.04 | n=12, r=-0.24, p=0.44 | n=12, r=0.38, p=0.23 | n=14, r=-0.39, p=0.17 | n=14, r=0.49, p=0.08 | n=14, r=-0.33, p=0.24 | n=14, r=0.52, p=0.05 |
| Paced tapping – MAD | n=11, r=0.37, p=0.26 | n=11, r=-0.29, p=0.38 | n=12, r=0.46, p=0.13 | n=12, r=-0.32, p=0.32 | n=15, r=0.19, p=0.51 | n=15, r=-0.07, p=0.8 | n=23, r=0.05, p=0.84 | n=23, r=-0.03, p=0.89 |
|  | n=11, r=-0.36, p=0.28 | n=11, r=0.73, p=0.01 | n=12, r=-0.23, p=0.47 | n=12, r=0.51, p=0.09 | n=15, r=-0.55, p=0.03 | n=15, r=0.78, p=0 | n=23, r=-0.29, p=0.18 | n=23, r=-0.03, p=0.9 |
| Visual reaction time – median | n=11, r=-0.55, p=0.09 | n=11, r=0.8, p=0 | n=13, r=-0.42, p=0.16 | n=13, r=0.54, p=0.06 | n=16, r=0.02, p=0.94 | n=16, r=-0.03, p=0.92 | n=25, r=-0.31, p=0.13 | n=25, r=0.12, p=0.56 |
| Visual reaction time – MAD | n=11, r=-0.15, p=0.67 | n=11, r=0.43, p=0.19 | n=13, r=-0.25, p=0.41 | n=13, r=0.57, p=0.04 | n=16, r=-0.18, p=0.51 | n=16, r=0.13, p=0.62 | n=25, r=-0.32, p=0.12 | n=25, r=0.13, p=0.53 |
| Decision time – median | n=11, r=0.13, p=0.71 | n=11, r=-0.09, p=0.8 | n=13, r=-0.54, p=0.06 | n=13, r=0.47, p=0.11 | n=16, r=0.01, p=0.96 | n=16, r=0.03, p=0.92 | n=23, r=-0.26, p=0.23 | n=23, r=0.27, p=0.2 |
| Decision time – MAD | n=11, r=0.27, p=0.42 | n=11, r=-0.28, p=0.4 | n=13, r=-0.49, p=0.09 | n=13, r=0.72, p=0.01 | n=16, r=-0.24, p=0.38 | n=16, r=0.39, p=0.14 | n=23, r=-0.43, p=0.04 | n=23, r=0.43, p=0.04 |
| Inhibition time – median | n=12, r=-0.26, p=0.42 | n=12, r=0.5, p=0.09 | n=14, r=0.28, p=0.33 | n=14, r=-0.2, p=0.48 | n=16, r=-0.34, p=0.19 | n=16, r=0.17, p=0.53 | n=23, r=-0.14, p=0.53 | n=23, r=-0.02, p=0.93 |
| Inhibition time – MAD | n=12, r=-0.14, p=0.67 | n=12, r=0.35, p=0.26 | n=14, r=0.04, p=0.89 | n=14, r=-0.46, p=0.09 | n=16, r=0.11, p=0.68 | n=16, r=-0.18, p=0.51 | n=23, r=0.13, p=0.57 | n=23, r=-0.13, p=0.55 |
| Neuro-QoL Cognitive Function | n=14, r=-0.06, p=0.83 | n=14, r=0.16, p=0.59 | n=16, r=0.17, p=0.52 | n=16, r=-0.33, p=0.21 | n=18, r=-0.02, p=0.94 | n=18, r=0.17, p=0.49 | n=26, r=0.16, p=0.43 | n=26, r=-0.08, p=0.71 |
| Neuro-QoL Depression | n=14, r=0.16, p=0.58 | n=14, r=0.19, p=0.52 | n=16, r=-0.38, p=0.14 | n=16, r=0.33, p=0.21 | n=18, r=-0.42, p=0.08 | n=18, r=0.47, p=0.05 | n=26, r=0.08, p=0.68 | n=26, r=-0.05, p=0.8 |
| Neuro-QoL Emotional and  Behavioral Dyscontrol | n=14, r=-0.12, p=0.68 | n=14, r=0.05, p=0.86 | n=16, r=-0.15, p=0.58 | n=16, r=0.1, p=0.7 | n=18, r=-0.53, p=0.03 | n=18, r=0.4, p=0.1 | n=26, r=0.18, p=0.37 | n=26, r=-0.18, p=0.38 |
| Neuro-QoL Fatigue | n=14, r=-0.13, p=0.66 | n=14, r=0.07, p=0.82 | n=16, r=-0.2, p=0.45 | n=16, r=0.26, p=0.34 | n=18, r=0.06, p=0.81 | n=18, r=0.04, p=0.89 | n=26, r=-0.01, p=0.94 | n=26, r=-0.06, p=0.79 |
| PROM-Ataxia psychosocial (Mental 1) | n=14, r=0.11, p=0.71 | n=14, r=-0.05, p=0.88 | n=15, r=-0.18, p=0.52 | n=15, r=0.37, p=0.18 | n=17, r=-0.51, p=0.04 | n=17, r=0.47, p=0.06 | n=25, r=-0.02, p=0.91 | n=25, r=-0.11, p=0.59 |
| PROM-Ataxia cognitive (Mental 2) | n=14, r=-0.01, p=0.97 | n=14, r=0.02, p=0.95 | n=15, r=-0.08, p=0.77 | n=15, r=0.31, p=0.27 | n=17, r=-0.49, p=0.04 | n=17, r=0.44, p=0.08 | n=25, r=-0.49, p=0.01 | n=25, r=0.39, p=0.05 |

***Note:*** Spearman correlations. Blue = small effect (0.1 < *r* < 0.3), orange = medium effect (0.3 < *r* < 0.5), purple = large effect (*r* > .5). CCAS-S = Cerebellar Cognitive Affective Syndrome Scale. Note that patterns of correlations across genotypes should be interpreted with caution due to small cohort sizes and non-equivalent participant characteristics between genotypes.
